# FASIMU: flexible software for flux-balance computation series in large metabolic networks

**DOI:** 10.1186/1471-2105-12-28

**Published:** 2011-01-22

**Authors:** Andreas Hoppe, Sabrina Hoffmann, Andreas Gerasch, Christoph Gille, Hermann-Georg Holzhütter

**Affiliations:** 1 Institute of Biochemistry, University Medicine Charité Berlin, Seestr. 73, 13347 Berlin, Germany; 2 Department of Parallel Computing, Wilhelm-Schickard Institute of Computer Science, Sand 13, 72076 Tübingen, Germany

## Abstract

**Background:**

Flux-balance analysis based on linear optimization is widely used to compute metabolic fluxes in large metabolic networks and gains increasingly importance in network curation and structural analysis. Thus, a computational tool flexible enough to realize a wide variety of FBA algorithms and able to handle batch series of flux-balance optimizations is of great benefit.

**Results:**

We present FASIMU, a command line oriented software for the computation of flux distributions using a variety of the most common FBA algorithms, including the first available implementation of (i) weighted flux minimization, (ii) fitness maximization for partially inhibited enzymes, and (iii) of the concentration-based thermodynamic feasibility constraint. It allows batch computation with varying objectives and constraints suited for network pruning, leak analysis, flux-variability analysis, and systematic probing of metabolic objectives for network curation. Input and output supports SBML. FASIMU can work with free (lp_solve and GLPK) or commercial solvers (CPLEX, LINDO). A new plugin (faBiNA) for BiNA allows to conveniently visualize calculated flux distributions. The platform-independent program is an open-source project, freely available under GNU public license at http://www.bioinformatics.org/fasimu including manual, tutorial, and plugins.

**Conclusions:**

We present a flux-balance optimization program whose main merits are the implementation of thermodynamics as a constraint, batch series of computations, free availability of sources, choice on various external solvers, and the flexibility on metabolic objectives and constraints.

## Background

The distribution of fluxes (i.e. reactions rates of enzymes and transporters) in large metabolic networks is commonly calculated by means of constraint-based optimization methods, usually referred to as flux-balance analysis (FBA). The first FBA applications relied on the steady-state assumption and biomass maximization only [1]. However, recently the scope of FBA has widened: (i) alternative objective functions are necessary to adapt FBA to different environments, metabolic excretion and growth patterns, and cell types [2,3], (ii) thermodynamic principles have to be considered in the calculation [4], (iii) mRNA, protein and metabolite profiles are available as an additional information source [5], (iv) metabolic networks are curated using on the fly verification [6], and (iv) FBA is used for structural network analysis avoiding the combinatorial explosion that topological algorithms suffer from. Thus, the quality of FBA software must now be measured on (i) the flexibility in the definition of objectives and constraints and (ii) the ability to perform batch series of calculations with varying objectives and constraints whose results are automatically evaluated.

Several software solutions for FBA are currently available. The freely available software COBRA [7] covers a considerable variety of FBA algorithms and is conceptually similar to FASIMU. It is a MATLAB [8] toolbox using a variety of free and commercial solvers including CPLEX via the TOMLAB^® ^Optimization Environment [9] as the recommended choice. OptFlux [10] covers an even larger range of flux optimization methods accessible through a graphical user interface. It is an easy-to-use solution well suited for biotechnologists with lesser interest in the algorithmic details. Systems Biology Research Tool (SBRT) [11] is a conceptually very powerful framework for the analysis of stoichiometric networks. A number of algorithms based on flux-balance optimizations is implemented and the addition of further "processes" is possible. The BioSPICE [12] framework includes two modules performing flux balance optimization: Fluxor [1] computes biomass maximization and MOMA [13] is the original implementation of the method with the same name. The open source program PathwayAnalyser [14] is a simple command-line program implementing FBA and MOMA. See Table 1 for a feature comparison of the cited packages.

**Table 1:** Feature Comparison.

	FASIMU	COBRA	OptFlux	SBRT	BioSPICE	Pathway Analyser
SBML import [41]	+	+	+	+	+	+
Flatfile import	+	+	+	+	+	-
SBML export [41]	+	+	+	+	-	-
expa export [45]	+	+	+	-	+	-
Metatool export [46,47]	+	-	+	+	-	-

Biomass maximization [1]	+	+	+	+	+	+
Flux minimization [29]	+	-	-	-	-	-
Regulatory network	-	-	-	+	-	-
MOMA [13]	+	-	+	-	+	+
ROOM [31]	+	-	+	-	-	-
Fitness maximization [30]	+	-	-	-	-	-
Expression profile match [18,19]	+	-	-	-	-	-
FVA [32-34]	+	+	+	+	-	-
MFA [35]	+	-	+	+	-	-
Thermodynamic feasiblity [17]	+	-	-	-	-	-

Batch computation & scripting	+	+	-	-	-	-
Description language	+	-	-	+	-	-
Graphical user interface	-	-	+	+	+	-
Not depend on commercial software	+	-	+	+	+	-
Solver selection	4	5	1	2	1	1
User documentation	+	+	+	+	-	-

Built-in visualization	-	-	+	-	-	-
Interaction with CellDesigner [42]	-	-	+	-	-	-
Interaction with Cytoscape [27]	+	-	+	-	-	-
Interaction with BiNA [26]	+	-	-	-	-	-

Feature comparison of recent programs focusing on flux-balance optimization. +/- denote the existance/absence of the feature. The row *solver selection *indicates the number of solver programs to choose from.

For all of the above programs, the use of thermodynamic feasibility as a direct constraint is currently missing. AnNET [15] is the only available tool so far that tests a given flux distribution on thermodynamic feasibility but only as a post-check and not as a constraint for FBA. A number of FBA-based algorithms such as pruning [16], thermodynamic realizability [17], inferring active subnetworks from expression profiles [18,19] are not yet available as an easy-to-use implementation. Batch processing of easily definable simulation series required for on-the-fly testing of network functions in a network curation process are not included in the above software solutions. This prompted us to develop a new software.

### Implementation

We have developed FASIMU - a comprehensive, flexible and user-friendly computation environment for FBA. Its command-line interface allows to tackle difficult problems in an interactive approach which can later be transferred into an executable computation script. FASIMU is "open" in two aspects: (i) the source code is open and written in widely known scripting languages which makes it easy to adapt and to implement new functions and (ii) intermediate results are stored in human-readable files rendering the calculation process traceable.

For the computational effort, FASIMU is divided in two parts. The computationally expensive part, the solution of the optimization problem, is left to specialized and highly optimized software: the commercials programs CPLEX [20] (currently freely available for academic purposes) or LINDO [21], alternatively the open source programs GLPK [22] or lp_solve [23]. The computationally easy but semantically complex part is written in a combination of scripting languages which are easy to understand and modify: the parser language gawk (GNU AWK) [24] and the script language bash [25]. bash is the default command-line shell in LINUX, MacOS, and many UNIX systems, so many computer users are familiar with it already. In FASIMU it is used to start and iterate gawk calls, define command-line functions, and to call the solver. gawk allows to program data processing in an extremely terse form. In FASIMU it is used to transform raw data into intermediate files, to prepare the input files for the solver, to interpret the solver output, and to generate result files. It is preinstalled on every LINUX and MacOS system and available for Windows and UNIX.

**Figure 1:**
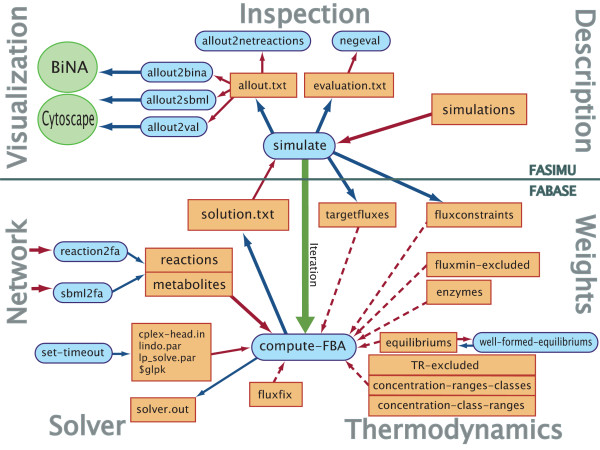
**FASIMU overview**. Overview on the functions and data files of FASIMU and the general layout of the software. Data files are shown in orange rectangles, function calls in blue rounded boxes, and external programs in green ellipses. Blue arrows refer to output data, red arrows to read data. The green arrow refers to an iterated function call. The bottom part refers to functions related to FASIMU, the top part refers to FASIMU. Dotted arrows refer to optional data. This table has been adopted and extended from [10].

FASIMU is structured in two layers: functions of basic layer, FABASE, deal with a *single *FBA problem, whereas functions of the upper layer, FASIMU, generate a series of FBA solutions by running FABASE functions iteratively (See Figure 1). The instructions for the iterative calls are listed in a user-editable file comprising (i) the simulation identifiers, (ii) metabolic target functions, (iii) constraints, and (iv) expressions for the automatic evaluation of the flux distribution. Therefore, in one such simulation series different metabolic objectives, enzyme knock-outs, and media composition can be considered. Upon a function call the simulations are performed and (i) an evaluation file as a short report on succeeded and failed computations and (ii) a detailed solution file are created. The latter can be further processed to provide the input files for BiNA [26], Cytoscape [27], or CellNetAnalyzer [28] visualization.

## Results

Algorithms implemented in FASIMU comprise biomass maximization [1], the principle of flux minimization [29], the fitness maximization at enzyme deficiencies [30], the minimization of metabolic adjustment (MOMA) [13] and the similar ROOM [31], inferring active subnetworks from expression profiles [18], also in combination with flux minimization [19], flux variability analysis [32-34], metabolic flux analysis [35], leak analysis [36], and pruning to the functional subnetwork [16]. It can be combined with constraints on flux values, metabolite exchange rates, and the thermodynamic feasibility constraint based on variable metabolite concentrations [17].

Crucial to the evaluation of a large number of flux distributions is their visualization. Here, we developed the plugin faBiNA for BiNA [26] showing a computed flux distribution where the thickness and color of reaction arrows visualize the flux rate. The compartment of the metabolite is either shown as a color of the metabolite node or all metabolites of a compartment are displayed in a separate box. The particular strength of BiNA is the customizable automatic generation of a graph layout using yFiles algorithms [37] which provide coherent graphs for up to 300 reactions. It can also be used to scroll through a set of precomputed flux distributions and show them in the context of the whole network or alternatively only the nonzero fluxes. The definition of reaction subsets (e.g. textbook pathways) will show the flux distribution in a hierarchical layout. Additional network information like gene expression can be mapped to node color or line thickness. Finally, flux modes can be exported by vector or scalar images. FASIMU also prepares the input files needed by CellNetAnalyzer [28] and FluxViz [38], a plugin for Cytoscape [27], especially suited to visualize the flux in the full network context.

## Example

In a network curation process the batch processing of defined simulations is required to verify metabolic functions on the fly. As an application example we show how FASIMU has successfully aided the curation of the genome-scale model of the human hepatocyte [39] (see tutorial chapter 3). The raw network as the output of the curation tool METANNOGEN [40] at the final stage comprised of 3369 reactions and 997 metabolites in 9 compartments (2458 localized metabolites). To assert the functional parts of the network the pruning algorithm [16] is applied yielding 2539 reactions and 777 metabolites (1420 localized). On a 64-bit LINUX PC (3 GHz processor) using CPLEX 10.1 the running time of this process was 27:37 min. As the process included 6411 single optimizations, that averages 0.26 s for a single optimization. On the reduced model we performed 442 simulations (defining the metabolic functions of the hepatocyte) which required 4:26 min by simple flux minimization, 0.6 s for a single optimization. The full computation including the thermodynamic feasibility constraint (can only be realized as a MILP problem) and a check on the computed solutions (a further optimization) required 10 h:43 min:44 s, 87 s for a single simulation. The protocol for these computations using (Additional file 1 saved as FASIMU_complete.zip) is:

unzip FASIMU_complete.zip

cd FASIMU_Liver_Example

sbml2fa liver.sbml

source fasimu

prune-network

cp MIMES.txt MIPES.txt PIPES.txt sub

cd sub

unzip ../../FASIMU_complete.zip\

FASIMU_Liver_Example/simulations

source fasimu

simulate

optimization_call="compute-FBA-T-c"

simulate

## Requirements

FASIMU can run on LINUX, Windows (from 98 or NT V ≥ 4 to the most recent Windows versions), MacOS, AIX, HP-UX and possibly many other operating systems since its minimal requirements, GNU bash [25], GNU awk [24] and GLPK [22] are open source and ported to many systems. FASIMU requires the alternative use of one of the solvers: the external solver lp_solve [23], GLPK [22], LINDO [21], CPLEX [20]. For Microsoft Windows, using Cygwin (Linux-like environment for Windows, http://www.cygwin.com) is recommended providing bash, gawk, GLPK for almost any Windows version available.

FASIMU's input is a stoichiometric model given in SBML [41], level 2 version 1-4, CellNetAnalyzer [28], or plain text format and additional text files specifying FBA objectives and constraints. FASIMU's computed flux distribution are returned in SBML level 2 version 4 or val files (plain text format compatible with CellNetAnalyzer [28] and FluxViz [38]).

## Discussion

With other powerful flux-balance optimization programs at hand, the question arises why another product is necessary. The fact that important published methods have not been available as an executable program has already been mentioned. An alternative to a new software would have been to implement the required algorithms in one of the more open programming frameworks COBRA [7], SBRT [11], BioSPICE [12] or even as direct plugins to Cytoscape [27], CellDesigner [42], SBW [43] or BiNA [26]. However, we found that the important preconditions could not be met in one of the solutions: (i) integration of powerful commercial solvers and free solvers, (ii) independence on the MATLAB framework, (iii) description language of simulations and the integration in scripts, (iv) easy implementation of new algorithmic ideas.

COBRA [7] has a comprehensive coverage of flux-balance methods, however, being a MATLAB [8] toolbox somewhat hampers its applicability. It is necessary to purchase a license of MATLAB for every machine COBRA should run. To use the best available LP solver, CPLEX [20], additionally a license of TOMLAB^® ^[9] is required. The modification and integration of COBRA into a workflow is confined to the MATLAB language and its API. In contrast, FASIMU can be integrated directly on the level of the operating system. In SBRT [11] the development of the mentioned algorithms as new processes is not as straightforward and it lacks the integration with a network visualization program. OptFlux [10] has quite a number of algorithms already implemented but lacks the flexibility of tools which are designed in a more open framework. The analysis of large networks is hampered by the fact that only GLPK [22] is used as the solver. In our comparison of the solvers in FASIMU we found that CPLEX is numerically stable for considerably larger MILP problems compared with GLPK. For BioSPICE [12], the development of flux-balance optimization is not the main focus in the BioSPICE development and only two algorithms are implemented. PathwayAnalyser [14] also covers only two FBA variants and its installation is not straightforward, it requires the solvers GLPK and OOQP http://pages.cs.wisc.edu/~swright/ooqp, the latter requiring BLAS and software from HSL [44] only available after registration and a FORTRAN 77 (not supported by recent gcc) compile process.

The main difference between FASIMU and all other comparable software is the concept of a concise description file for flux-balance simulations. Its development has been driven by the necessity of a clearly defined protocol for network testing. The main objective was that the description file contained the minimal necessary information but allows considerable flexibility to define heterogeneous network tasks, beside (i) the simple test on the producibility or degradability of metabolites, also (ii) simulations of enzymopathies, (iii) tests on the non-existence of solutions, and (iv) tests on side conditions in the flux distributions. For instance the 442 simulations to test HepatoNet1 [39] are contained in a text file of only 57 k characters (additionally 3 interface description files of 1227 characters). This is extremely condensed given that it even contains some documentation.

A freely available software based on free and widely available software has large advantages for a first test as the program can immediately be tested. Aside from the consideration whether the fee for a commercial product such as MATLAB or CPLEX is worth the investment free software such as GNU bash, gawk, and GLPK is available for the maximum of possible computer architectures and operating systems.

Basing FASIMU on free software is a practical consideration rather than a decision on principle. We found out that for problems such as a feasible MILP implementation for a large metabolic system, the available free software is not yet sufficient. Thus, to integrate CPLEX or LINDO is a logical consequence of common sense: *free software where possible, commercial where necessary*. The same is true for the use of BiNA which uses powerful algorithms to draw network graphs. BiNA is freely available but not open source since it uses the commercial software yFiles licensed for free of charge use in conjunction with BiNA.

For FASIMU a compilable language such as JAVA or C++ was not used to support easy modification. FASIMU is also designed as a testing environment for newly developed algorithms, thus, development time is critical. Furthermore, the majority of the computation time is used by the external solver program. Therefore, to decrease the running time of the software which merely controls and transforms the input and output of the solver program would not have a great effect.

Graphical user interfaces (GUI) allow an easy access to the possible options and require minimal learning time. However, users who want to combine and modify the given algorithms rely on a scripting language. Programs allowing both the control by a graphical user interface and by a command-line interface require considerably more software development time. For FASIMU the focus is clearly the command-line usability. Powerful network visualization products such as BiNA, Cytoscape, CellDesigner, SBW [43] have already been developed. Thus, the integration of FASIMU with the programs mentioned appeared to be the better solution than the development of a separate visualization component in FASIMU. This integration is simplified by the SBML standard [41]. FASIMU solutions can be converted to SBML. However, to increase the usability in connection with FASIMU computations, faBiNA and FluxViz have been developed to allow an even better control of BiNA and Cytoscape.

## Conclusion

We present a Flux Analysis SIMUlation framework which (i) offers the first available implementation of thermodynamic feasibility as a quickly computable MILP, (ii) is flexible in the choice of objective functions and constraints, (iii) allows for batch processing of heterogeneous computations and automatic evaluation of the solutions, (iv) facilitate visualization of the computed fluxes with plugins for BiNA and Cytoscape, and (v) can completely be based on free software.

## Availability and requirements

**Project name **FASIMU

**Project home page **http://www.bioinformatics.org/fasimu

**Operating system(s) **Platform independent.

**Programming language(s) **bash version 3.0.0 or higher, version 4.0.0 or higher recommended. gawk version 3.0.0 or higher.

**Other requirements **Any of CPLEX (version 9-12 tested), GLPK (version 4.42 tested), lp_solve (version 5.5 tested), LINDO (version 5.0.1.317 tested).

**License **GNU GPL

**Any restrictions to use by non-academics **none.

## Authors' contributions

AH developed FASIMU; SH, AG, and AH wrote faBiNA. AH and CG wrote the manual/tutorial. AH, SH, HH, and CG drafted the manuscript, approved by all authors.

## Supplementary Material

Additional file 1 **FASIMU 2.2.1 release archive**. This archive contains the complete FASIMU distributions and unzips in five directories: **FASIMU **contains the programs, **FASIMU_-_Doc **contains the documentation (manual and tutorial), **FASIMU_-_Ery_-_Example **contains a small example of the human erythrocyte, **FASIM_-_Ecoli_-_Example **a large example of the *E. coli*, **FASIMU_-_Liver_-_Example **another large example of the human hepatocyte. Click here for file
