# Supplementary material for: FASIMU: flexible software for flux-balance computation series in large metabolic networks
Source: BMC Bioinformatics. 2011 Jan 22;12:28. doi: 10.1186/1471-2105-12-28 (PMC3038154; doi:10.1186/1471-2105-12-28)
Supplement: Additional file 1 — FASIMU 2.2.1 release archive. This archive contains the complete FASIMU distributions and unzips in five directories: FASIMU contains the programs, FASIMU-Doc contains the documentation (manual and tutorial), FASIMU-Ery-Example contains a small example of the human erythrocyte, FASIM-Ecoli-Example a large example of the E. coli, FASIMU-Liver-Example another large example of the human hepatocyte. [file 1471-2105-12-28-S1.ZIP › FASIMU_Doc/FASIMU_Manual.pdf]

Manual FASIMU

Andreas Hoppe - hoppe@bioinfomatics.org

November 25, 2010

This manual documents version 2.2.1.

Chapter 1

Introduction

FASIMU is an extremely flexible computation environment for different sorts of flux-balance analysis. The flexibility is traded off with simplicity of use. However, people who are acquainted with command-line interfaces will find it easy to use. On the pro side is that practically any algorithmic parameter can be adjusted and it is therefore especially suited for the scientific workflow: difficult problems can be tackled interactively and session protocols can be used to implement new flux-balance methods.

FASIMU is very open in two aspects: The source code is open and written in a high-level language which makes it easy to adapt and implement new functions. The other aspect is that intermediate results are stored in human-readable files which can easily be analyzed once the internal structure of the workflow is understood.

The interactive language used for FASIMU is one that most LINUX and MacOS users along with many other UNIX and Windows users are familiar with: the Bourne again shell (bash) [1]. Thus, anything working in bash can easily combined with FASIMU. The typical FASIMU session is in every respect identical to the interactive work in bash. In other words, FASIMU consists of available bash-functions. This concept is more flexible than the interactive languages of Mathematica or MATLAB as the integration with other programs is much more straightforward. For users of LINUX the learning curve is very steep for the syntax of the available commands. Writing algorithmic ideas based on FASIMU is technically writing additional bash functions. Programs in other languages can this way easily be combined with FASIMU; the only prerequisite is an executable program and a defined file exchange.

In flux balance analysis by far the most computational expensive part is the work of the optimizer. Thus, it is not much of an advantage to write the control of the algorithm in a low-level language. This is the reason that the implementation of FASIMU is consequently divided into two parts: the computationally difficult part is handled with an external solver (currently CPLEX, LINDO, lp_solve, or GLPK), the computationally easy but semantically complex part is written in a high-level language which is easiest to understand and modify.

1.1 Layer-structure

FASIMU consists of a two-layer structure: the outer layer is FASIMU itself and the lower layer is called FABASE. Where FABASE is focused on a single flux optimization, FASIMU supplies the iteration of flux optimizations with FABASE. FABASE can be used on its own when only single flux computations are required, FASIMU depends on FABASE. It is worth to keep in mind which function belongs to which level. The crucial item of the interface between the two layers is the shell variable "`$optimization_call`". It contains a FABASE function with full parameters which is called for any of the FASIMU functions which iterate flux calculations.

There is also another respect in which FASIMU sets a layer structure: on the metabolic model itself.

When initially called, FASIMU modifies the model by setting a new boundary around the system and the addition of virtual exchange processes for any metabolite of the system across this boundary. The result is another FABASE-compatible model but with a few special properties. Usually, this works unattended in the background but it appears in the computed flux solutions. The duplicated metabolites can be recognized at the "`_ex`" ending. The virtual exchange processes hold a "`_tr`" suffix.

1.2 The logical structure of a FASIMU session

A FASIMU session should be started in a single empty directory. This directory will later contain the various input, output, and intermediate files of this particular session. For the analysis and proper reference it is advisable not to mix files from different sessions or edit the files in this place. FASIMU will probably work anyway but it is more difficult to assess if it is really doing what the scientist intended to do.

- The first step is to copy all files related to the metabolic network into that directory. It is advisable to store the information to one model version in a separate directory. The metabolic model must be supplied in FA-Format, this is a proprietary extended version of CellNetAnalyzer-Format. There are transformation tools available for SBML and a simple Reaction-Scheme-Format. See chapter 3.
- The second step is the call of "`source fasimu`" which has several purposes. It transforms to model into a form compatible with FASIMU and makes the FASIMU functions available. Then it starts FABASE which supplies the FASIMU functions and writes some intermediate files which accelerate the preparation time for a single flux optimization call: the main parts of the input into the optimizer program is the prepared (in LPF-Files). See section 4.1.
- The third step (which also might be interchanged with the second) is the writing of the iteration scheme, a file with the fixed name "`simulations.txt`". One line defines a single flux computation. See section 4.2.
- The fourth step is setting the variable "`$optimization_call`" which defines the algorithm of the flux computation. See section 4.3.
- The fifth step is the start of the simulations with "`simulate`". This function proceeds with all simulations, there are also functions which select a simulation etc. See section 4.4.
- The sixth step is to review the file "`evaluation.txt`" whether the reported results have been satisfactory. There is one line for each simulation holding a "+" if it obtained the desired result, "–" otherwise. See section 4.5.
- The seventh step is to view the flux distributions stored for all solutions in a file "`allout.txt`". Single flux distributions are selected with "`allout-select <name of the simulation>`". The I/O-fluxes are at the beginning and should be reviewed first whether the system boundary fluxes are as desired. See section 4.6.
- The eighth step is advisable if the flux distribution seems reasonable but too complex to check in text form: the visualization in BiNA [13], Cytoscape [11], or CellNetAnalyzer [12]. Basically there is the function "`allout2bina`" which creates directories of input files for the flux analysis plugin of BiNA, or the function `allout2valfiles`. See section 4.7.

All these steps are described in greater detail below.

1.3 General guidelines of FASIMU's user interface

Fixed file names

The model under investigation be FASIMU resides in **fixed file names**. This has the immediate consequence that in one directory there can only be one model at a time.

Shell variables

Some information related to the present model are stored in local variable names. It is therefore not possible to change the directory to another FASIMU model and continue there. It is necessary to call `source fasimu` again in this new directory. This call regenerates the contents of the variables `$rea`, `$met`.

Some variables are set to default values by `source fasimu` if they are not defined but the are not overwritten if they are already defined: `$optimzation_call`, `$default_compartment`, `$solver`, `$names_in_allout`.

File contents

All input files are whitespace-separated text files except for the file `simulations` where a two level separation is necessary: the primary separator is the tab and the secondary separator are space characters. FASIMU's structured main output files (`allout.txt` and `evaluation.txt`) are tab-separated. FABASE output files are whitespace separated.

File extensions

All files to be supplied by the user are text files with a specific name such as `reactions` without an extension. Some of these files are modified during the course of computation, their original content is kept in `<file-name>.original`. All files produced by FASIMU being of interest to the user have the extension `txt` or `out`. Several files are produced during the workflow. They are recognized by their extension:

Extension	Function
none	input file
`original`	backup of input files
`txt`	produced by FASIMU being of interest to the user
`fgf`	internally used by FASIMU
`lpf`	parts of the problem formulation
`lp`	CPLEX-LP format
`ltx`	LINDO-LP format
`par`	parameter files of LINDO and lp_solve
`in`	Input to external programs
`out`	Output of external programs and extracts thereof

Function names

Function names carry dashes and not underline characters (except if it contains the identifier lp_solve which is dictated by the name of the solver)

File parameter

Functions do not accept file parameters, instead the files used have fixed names. If several files for the same purpose are used throughout a session, the session script must rename them. Few exceptions are `compute-moma` and `compute-room` which take a reference soution as a parameter.

Switch function upon file existence

Many functions are switched on if the respective file exists: e.g. fluxes are fixed if the file `setfluxes` just exists. Some files are used only for certain algorithms, such as `fluxmin-weights` is used only for flux minimization. Other files are only used if explicitely switched on such as `expressions`.

Switch functions upon file existence	
used for all optimizations	`fluxbounds` `setfluxes` `fluxfix`
used for flux minimization	`fluxmin-excluded` `fluxmin-weights`
used for thermodynamic constraint	`TR-excluded` `concentration-class-ranges` `concentration-ranges-classes`
must be switched explicitely	`expressions` `enzymes`
only in FABASE session	`fluxconstraints` `targetfluxes`

File overwrite

As a consequence of fixed file names, the invocation of FASIMU with `source fasimu` changes the input files for FABASE: `reactions`, `metabolites`, `equilibriums`, `targetfluxes`. The original files are kept in respective files with the extension `.original`. This concept has historical reasons (FABASE was first, FASIMU has been set on top of it). The parameter files (`cplex-head.in` and files with the extension `par`) are only created by `source fabase / fasimu` if not present. Present files will only be overwritten if explicitly requested by the `restore-default-parameters` function. Also, the function `set-timeout` modifies these parameter files.

Effects of source fasimu	
`reactions`	modified, backup in `reactions.original`
`metabolites`	modified, backup in `metabolites.original`
`equilibriums`	modified, backup in `equilibriums.original`
`fluxmin-excluded`	modified, backup in `fluxmin-excluded.original`
`TR-excluded`	modified, backup in `TR-excluded.original`
`concentration-ranges-classes`	modified, backup in `concentration-ranges-classes.original`

During the FASIMU functions `simulate` and `simulate-single`, the files `targetfluxes` and `fluxconstraints` are overwritten and should not be modified by the user. In a FASIMU session, `setfluxes` and `fluxbounds` should be used instead.

Choice FASIMU vs. FABASE session

By starting the session with

```
source fasimu
```

you have all features of FASIMU but you can also start the so-called FABASE session with

```
source fabase
```

which give more manual control options. See chapter 6 (Using FABASE without FASIMU).

Chapter 2

Installation

FASIMU requires the basic UNIX tools bash and gawk and an optimization solver. Also, a visualization package increases its usefulness.

2.1 Bash and GNU awk

On standard computer systems like UNIX, LINUX and Macintosh, bash and gawk (GNU awk) are installed as part of the operating system. On UNIX systems, where these tools are not yet available, they can be compiled from the freely available source code, see http://www.gnu.org/software/bash and http://www.gnu.org/software/gawk. The version of bash should be at least 4.0 for the function `prune-network` to work, the rest of FASIMU also works for older versions.

GNU AWK is an implementation of a more generally defined language AWK and has some extensions. FASIMU uses some of the extensions, thus, other implementations of AWK might not work for FASIMU. Therefore it is not a good practice to link or rename gawk to awk. FASIMU calls gawk explicitely. Call

```
gawk -W version
```

from the command line to check if that works.

The actual position of bash and gawk in the file system may vary in certain UNIX distributions. FASIMU assumes `/usr/bin` as the position of gawk but it may also be `/bin`. If that is the case, the script `sbml2fa` and `reaction2fa` may result in an Error message "`/usr/bin/gawk not found`". The easiest way then is to call it with:

```
gawk -f /usr/local/bin/sbml2fa <SBML-file>
```

if the install directory of FASIMU is `/usr/local/bin`. This is the only issue related to the file position. However, it is obviously necessary that gawk is in the search path (variable `$PATH`).

FASIMU on Windows

On Microsoft Windows systems bash and gawk are usually not installed. They can be conveniently installed by installing Cygwin. The recent version of Cygwin supports, Windows NT from version 4.0, Windows 2000, Windows XP, Windows Server 2003, Windows Server 2008, Windows Vista, Windows 7. Install a cygwin system by the calling the URL http://www.cygwin.com/setup.exe in a browser window and execute the setup program. I the process of installing you are asked to enter a directory to install cygwin in and a mirror to download software. Finally, you get a window to select the software to install. You can check whether gawk and bash are on the list of the software to install but that should normally be the case. You should also install glpk (in the Math directory), simply enter glpk in the search box and click to select for install.

On Windows 98, Windows ME, Windows CE an oder version of cygwin can be installed, use the URL http://www.cygwin.com/setup-legacy.exe to install.

After the installation a program Cygwin bash shell is added to the start menu. Start that and you are in bash shell and can proceed to install FASIMU.

2.2 Further useful tools for FASIMU

The UNIX tools `wget`, `unzip` (GNU zip), `diff`, `wc`, `less` and `grep` are normally also installed on any system where also `bash` is installed. They are useful for the command-line work and are occasionally used through out the examples in the manual and the tutorial.

The http-program `wget` is used to download FASIMU files from the bionformatics.org server. If you are protected by a firewall program which connects through the internet with a proxy, you have to set the variable `http_proxy`.

2.3 Install FASIMU

The starting point for the below commands is a running bash shell. If the current shell is not bash (see the variable `$SHELL`) problems might occur. This can easily be solved by calling

```
bash
```

Unzip `FASIMU.zip` in a directory contained in the search path. Following UNIX conventions, the directory `/usr/local/bin` may be used but you normally have to have super user attributes to write there:

```
cd /usr/local/bin
wget http://www.bioinformatics.org/fasimu/FASIMU.zip
unzip FASIMU.zip
rm FASIMU.zip
```

Alternatively, any directory such as `~/bin/` may be used. In this case the `PATH` variable should be extended by including the following line into the the start-up file `~/.bashrc` of the command line interpreter:

```
   export PATH="$PATH:~/bin"
```

2.4 Optimization solver

FASIMU was originally developed with the commercial solver ILOG CPLEX, but also the free solvers LP_SOLVE and GLPK can be used, additionally the LINDO API solver. However, some FASIMU functionality (those involving quadractic objectives and constraints) is not available with these alternative solvers.

CPLEX

CPLEX is a commercially available solver (see http://www.ibm.com/software/integration/optimization/cplex-optimizer). FASIMU is tested with versions 9, 10, and 12.2 but other versions should also work. There is a highly relevant difference between these two versions. IBM offers the recent version 12 free of charge for academic users, see http://www.ibm.com/developerworks/university/academicinitiative/ for more information.

FASIMU uses the interactive solver of CPLEX which is called "`cplex`" and must be in the search PATH. If this is not already the case, there are two options to do this. Either, the directory containing the cplex executable can be added to the PATH, e.g.:

```
export PATH=$PATH:/usr/ilog/cplex101/bin/x86-64_RHEL3.0_3.2/
```

Second option is to add a symbolic link in a directory in the search path, e.g.

```
ln -s /usr/ilog/cplex101/bin/x86-64_RHEL3.0_3.2/cplex /usr/local/bin/cplex
```

LINDO API

FASIMU uses the interactive solver of LINDO API which is called "`runlindo`" and must be in the search path. If this is not already the case, there are two options to do this. Either, the directory containing the runlindo executable can be added to the PATH, e.g.:

```
export PATH=$PATH:/usr/local/lindoapi/bin/linux64/
```

Second option is to add a symbolic link in a directory in the search path, e.g.

```
ln -s /usr/local/lindoapi/bin/linux64/runlindo /usr/local/bin/runlindo
```

GLPK

GLPK is an open-source free solver (see http://www.gnu.org/software/glpk/) which is available for LINUX and Windows. It is ported to LINUX gentoo and is probably part of other major LINUX distributions. It is ported to cygwin and can therefore the easiest way to run FASIMU under Windows. FASIMU uses its interactive solver which is called "`glpsol`" and must be in the search path which should already be the case if it is installed as part of a LINUS distribution or cygwin.

lp_solve

lp_solve is another open-source free solver (see http://lpsolve.sourceforge.net/) which is available for LINUX and Windows. It is ported to LINUX gentoo and is probably part of any major LINUX distribution. lp_solve is tested in the version 5.5.

FASIMU uses its interactive solver which is called "`lp_solve`" which must be in the search path. It easiest to move the executable in `/usr/local/bin` which is the default installation. lp_solve requires the filter libxli_CPLEX.so which is included in the lp_solve distribution and should preferentially be placed in `/usr/local/lib`.

2.5 Visualization packages

BiNA

Download the latest version from http://sourceforge.net/projects/binafluxmodel which includes BiNA and the preinstalled flux analysis plugin. Set the shell variable `BINA_HOME` to the directory the file `BiNA.jar` is located in.

Cytoscape

Download the latest version from http://www.cytoscape.org and install as documented at the side. Install the plugin FluxViz as documented in http://www.charite.de/sysbio/people/koenig/software/fluxviz/help/index.html#Installation

CellNetAnalyzer

CellNetDesigner is a commercial network analysis tool free for academic use, see http://www.mpi-magdeburg.mpg.de/projects/cna/cna.html. Although graphical output is not the specific purpose of this program, a combined use with FASIMU has some advantages.

The internal data format (the files `reactions` and `metabolites`) is identical in FASIMU and CellNetAnalyzer. Thus, it easy to transform a model developed with CellNetAnalyzer in FASIMU. The feedback of computed flux modes is easy.

Chapter 3

FABASE-models

3.1 Getting a model from CellNetAnalyzer

The format of the files `reactions` and `metabolites` equals the internal format of CellNetAnalyzer, formerly FluxAnalyzer. Some parameters in these files have no meaning inside FABASE and are retained for compatibility only. Thus, a model developed with CellNetAnalyzer is a good starting point for a FABASE model. Computed flux modes can easily be transferred to CellNetAnalyzer since the format of the `.val` files is also compatible.

3.2 Getting a model from SBML

The easiest way to produce a FABASE model is to get or produce an SBML file and use the program sbml2fa which comes along FASIMU. It is capable to read SBML2.1 to SBML2.4 as it only reads the reaction network which did not change in the subversions. For the metabolites it uses the tags: id, name, compartment, boundaryCondition. The latter three may be missing, after a warning a default is set. For the reactions the tags id, name, and reversible are being used, the latter two might be missing. Additionally, a non-standard way to encode the equilibrium constant will be read:

```
<listOfParameters>
  <parameter id="equilibrium_constant" value="...">
</listOfParameters>.
```

There are some issues where the SBML definition is not compatible with the definition of a FASIMU model. The first issue is that FASIMU encodes the compartment in a suffix to the metabolite identifier and name headed by the underscore character. Therefore underscore characters are not allowed in the compartment identifier (there are no problem in the metabolite names however). sbml2fa removes the underscore characters and also the space characters from the compartment names. If different compartments carry identifiers which differ only by some underscore or space characters (definitely not a sensible practice!) sbml2fa merges them to one. The second issue is closely related. If the metabolite identifier does not end with a `_<compartment>` already it is added. The same is done independently with metabolite names.

The second issue is that SBML allows space characters in metabolite names, but FASIMU does not (in fact, metabolite name serve as alternative identifiers in the simulation file). Therefore, sbml2fam replaces space characters by underscore characters in metabolite names.

Both issues result in the fact that SBMLs generated with FASIMU may have not the same identifiers as the input SBML. But if the following conditions are met, the SBML is FASIMU-proof, meaning SBMLs generated from FASIMU are compatible with the original SBML given to sbml2fa:

- Compartment identifiers do not contain underscore characters

- Metabolite identifiers do not contain space characters
- Metabolite identifiers end with `_` and the compartment identifier
- Metabolite names end with `_` and the compartment identifier

3.3 Getting a model from a reaction scheme

Another simple way to get a basic FABASE model is to use the program reaction2fa which also comes along FASIMU. It is fairly robust to read any text format reaction scheme which is available in the literature, e.g. the reaction schemes the earlier Palsson group publications uses. The basic format is a whitespace separated file where each line describes a single reaction. It starts with the reaction identifier, than the reaction using numbers (for the stoichiometric coefficients, 1 can be omitted), the identifiers of metabolites, the + sign and reaction arrows as "`<=>`" and "`-->`" resp. "`<--`" for irreversible reactions. Neither the names of metabolites nor the boundary condition of metabolites can be set this way. This must be done with modifications of the `reactions` and `metabolites` file.

3.4 Writing a FABASE model from the scratch

This is easier than it might seem. For a basic FABASE model only two files are necessary, "`reactions`" and "`metabolites`" which are plain, white space separated text files. The elements surrounded by the white space (arbitrary sequences of space and tabulator characters) are called token.

Metabolites: `metabolites`

The first token is the metabolite identifier which must be unique. The next token is the name which must not contain white space. Typically, space characters are replaced with underscore characters. The next token is arbitrary for the use within FABASE. The next token is the boundary condition, 1 for metabolites which may freely enter or leave the system and 0 for all other metabolites. At this point it must be noted that the boundary condition at this point is only used for direct FABASE calculations (which are not described in this manual) and is ignored for the use in FASIMU: the boundary condition is controlled in the simulations.txt file.

For the use in FASIMU it is necessary to mark the compartment (consisting of letters only) as a suffix separated with a "`_`" character. Only for working with FABASE without FASIMU model they are not considered.

It is recommended to mark the names of the metabolites with the same compartment suffix. This allows to use metabolite names in the `simulations` file. If the names do not have correct suffixes the FASIMU function `simulations-work-assure` (called by `simulate`) will warn about them.

Reactions: `reactions`

The first token is the reaction identifier which must be unique. The next tokens describe the reaction, where stoichiometric coefficients are obligatory and the reaction arrow is strictly =. After the reaction two fixed tokens follow: "`|`" and "`#`". The next two tokens refer to the flux bounds, the first one for the lower, the second one to the higher. "`-Inf`" is legal for the lower, "`Inf`" for the upper bound. To set the reversibility of a reaction, it must be set in the flux bound, there is no indicative reaction arrow.

3.5 Refining the FABASE Model

The following files are whitespace separated.

Equilibrium constants: `equilibriums`

In FASIMU equilibrium constants are used as weights for the backward fluxes in the flux minimization [5] and to implement the thermodyamic feasiblity constraint [8]. The Gibb's free energies can not be given directly, compute the equilibrium constants with the formula:

$$\mathrm{K_{eq}} = \mathrm{e}^{-\frac{\Delta \mathrm{G_r^0}}{RT}}$$

where R is the universal gas constant and T is the absolute temperature. As you can see from the formula, a FASIMU model has a fixed temperature.

Note that the equilibrium constants for FASIMU are the most frequently used dimensionless constants related to standard concentrations of 1M (not of 1mM as sometimes used).

The first item in every line of the file `equilibriums` is the reaction identifier, the second is the equilibrium constant. If the equilibrium constant of a reaction is not explicitely given it is assumed to be 1. Therfore, to exclude a reaction from thermodynamic consideration it is not sufficent not to give the equilibrium constant, it mus explicitely mentioned in `TR-exclude`, likewise in `fluxmin-exclude` to exclude it from the flux minimization scoring function.

Set fluxes to specific values: `setfluxes`

This (white-space separated) file sets fluxes to specified values. The first token of each line is the reaction identifier. If it is the only token of this line the respective flux is fixed to zero. If one token follows the fluxed is fixed to this value. This feature is used if the file with the name exists. Take care to delete or rename the file if it no longer needed.

There are two differences to the file `targetfluxes` described below: (i) it is not modified by functions of FASIMU and (ii) the fluxes are always fixed to the respective values whereas there are not strictly set to the `targetfluxes` when the fitness maximization feature is used.

There is no difference to `fluxfix` when it is only used to fix it to values and not to other fluxes. The `setfluxes` overwrites the value given by `fluxfix`.

Set boundaries for fluxes: `fluxbounds`

This (white-space separated) file sets flux boundaries in conjunction to the values set in `reactions`. The first token of each line is the reaction identifier. If it is the only token of this line the respective flux is fixed to zero. If one token follows the absolute value of the flux must be lower than the value of this token. I two tokens follow they represent the lower and upper bound. "`Inf`" and "`-Inf`" are allowed, representing unrestricted fluxes.

In conjunction with other bounds is interpreted in the normal way: the maximum of all applicable lower bounds is the lower bound and the minimum of all upper bounds is the upper bound.

This feature is used if the file with the name exists. Take care to delete or rename the file if it no longer needed.

Fix fluxes to a linear combination of others: `fluxfix`

It is possible to set that a particular reaction flux is fixed to a linear combination of other fluxes. Such reactions can also be reformulated but for clarity of the network it might be useful to retain them as separate reactions and fix them separately. An example is that 3

`fluxfix` is a white-space separated file. The first token is the flux to fix. The rest of the line represents the linear combination, similar to the "`enzyme`" file: First the coefficient, then the reaction identifier. The coefficient can be omitted in which case it is assumed to be one. The reaction identifier can be omitted for the last token, which means that this value is added as a direct value. Thus, the function can also be used to fix reactions to a preset value.

This feature is used if the file with the name exists. Take care to delete or rename the file if it no longer needed.

Exclude reactions from the cost function: `fluxmin-excluded`

In the different computations based on flux minimization every reaction is used for the objective function. Including reactions in “`fluxmin-excluded`” is an easy way to exclude certain reactions (e. g. those which do not describe a cellular effort).

Set relative weights for the cost function: `fluxmin-weights`

By default, every flus has the same relative weight in the scoring functions in the flux minimization principle. The file `fluxmin-weights` however, changes the relative impact of individual functions. Not all reactions have to mentioned in this file, in this case 1 is assumed. Also the weight 0 can be given, it has the same effect as including the reaction in `fluxmin-excluded`. The function `fluxmin-excluded` takes precedence over `fluxmin-weights`, that means if a nonzero weight is given in `fluxmin-weights` but is also mentioned in `fluxmin-excluded` then the reaction is excluded from the score calculation.

Concentration ranges

Concentration ranges must be supplied if TR-modes should be computed. To simplify specification of concentration ranges, concentrations may be given for classes of metabolites rather than for metabolites directly. The file `concentration-ranges-classes` assigns a metabolite to a class.

The file `concentration-class-ranges` then assigns concentration ranges to classes. Each line usually contains 6 tokens: The class identifier, the lower hard bound, the lower soft bound, the set point, the upper soft bound, and the upper hard bound. The hard bounds must never be exceeded. The soft bounds may be violated at the expense of an additional penalty. If a concentration value differs from its set point, a (usually low) penalty is given in the objective function (if this function in switched on). Depending on the number of tokens the following rules apply:

Number of values	Ranges definition	Applied assignments
5	`<low hard> <low soft> <setpoint> <high soft> <high hard>`	
4	`<low hard> <low soft> <high soft> <high hard>`	`<setpoint>`= $\sqrt{< \text{lowsoft} >< \text{highsoft} >}$
3	`<low hard> <setpoint> <high hard>`	`<low soft>=<low hard>`,<br>`<high soft>=<high hard>`
2	`<low hard> <high hard>`	`<setpoint>`= $\sqrt{< \text{lowsoft} >< \text{highsoft} >}$,<br>`<low soft>=<low hard>`,<br>`<high soft>=<high hard>`
1	`<fixed concentration>`	

For most applications (when no concentration prediction is required) only the hard bounds are required. For the soft bounds and the set points there is also allowed to write a minus sign instead of a value. This is interpreted as there is no boundary for this particular metabolite class. Also it is possible to assign a higher importance to particular set points or soft bounds:

```
<value>(<weight>)
```

where the default weight is 1.

Exclude reactions from TR-computations: `TR-exclude`

Thermodynamic feasibility can only be assessed if the standard Gibb’s free energies of a reaction is known. Therefore FASIMU/FABASE allows to selectively exclude reactions from the TR constraint: put the identi-

fier of the reaction in the file `TR-exclude`. Often, Gibb's energies of reactions are computed as the difference of formation energies of products and reactants. If a formation energy is not known, the Gibb's energy of all reactions involving this metabolite is presumably also not known. Thus, another option is to enter a metabolite identifier in TR-exclude: this means that any reaction involving this metabolite is excluded from the TR criterion. Note that the identifier must include the compartment suffix. This is a consequence that TR resides at the FABASE-level which (as opposed to FASIMU) does not know about compartments. TR-excluded is a white-space separated file, the tokens are accepted no matter if they appear in the same line or in different lines.

Additional names of reactions: `reaction-names`

This is a white-space separated file: the first token on each line must be a reaction identifier, the rest of the line is regarded as the name of the reaction. The name of the reaction is for instance added at the end of each reaction flux line in allout.txt.

Enzymes and specific weights: `enzymes`

In basic flux minimization every (forward) flux is considered to be of equal importance, i.e. the optimization function is the sum of (absolute values of) fluxes. However it is also possible that the fluxes have different weights in the optimization. In the FA-model they are written in the file "`enzymes`". The name of the file is derived from the most straightforward use of this function: to implement a cost-minimizing principle of enzymes. The syntax is as follows. It is a white-space separated file. The first token on each line is the identifier of the enzyme. It is no problem if that identifier also appears as a reaction identifier, enzymes and reactions have different name spaces. Then, a coefficient follows, then a reaction identifier. The semantics is that the enzyme requires the cost (the coefficient) to catalyze a unit flux through that specific reaction. The coefficient can also be omitted, in which case it is considered to be 1. The coefficient zero is also allowed, it means that the respective reaction is assigned no cost with respect to this enzyme.

Several important notes for this function: 1. As opposed to other files in the FA-model it is not automatically used, the respective optimization function must require this: e.g. "`compute-FBA -F E`". 2. A reaction which is not referred to in "`enzymes`" is considered to have no cost, it is not minimized. 3. The information is only used for flux minimization computing scripts, other algorithms (biomass maximization or intake minimization= do not use it. 4. The use of this function requires that the reaction identifiers may must not consist only of digits, it is clear that they can be confused with the coefficients.

FABASE files used to implement FASIMU: `targetfluxes` and `fluxconstraints`

The file "`targetfluxes`" provides a way to fix the flux rate of a reaction (first token) to a value (second token). The file "`fluxconstraints`" borders the flux value. An upper limit is given as one token, whereas a range requires two tokens. The simulation objective (see `simulations` below) is implemented with `targetfluxes`, the simulation constraints are implemented with `fluxconstraints`. Since FASIMU automatically generates these files prior invocation of a FABASE function, these options are only available when FABASE is used without FASIMU (see below).

3.6 FASIMU-compatible Models

For compatibility, conventions of CellNetAnalyzer have been adopted for FABASE and FASIMU:

- Space characters are not allowed in identifiers.
- Reaction identifiers may be not by number number.

- Every metabolite must have an assigned compartment where the compartment is attached as suffix to the metabolite identifier preceded by underscore.
- Compartment identifiers must consist only of letters.
- The compartment "`ex`" is not allowed.

3.7 Modify model files in a FASIMU session

The recommended way to handle your models

Generally, I do not recommend to modify the files in the directory in which you are working with FASIMU. The program may be capable to regenerate intermediate files and for advanced users it makes sense to do so (see below). But to start with I strongly recommend that you keep the original models in separate directories and start fasimu in a fresh directory. Say, you keep and modify your model in a directory `~/model` then proceed like that:

```
cd ~
cp -R model model-compute
cd model-compute
source fasimu
<do the computations>
<copy the result files to a designated directory>
cd ..
rm -r model-compute
```

If you are acquainted with FASIMU you will certainly design your own work flow.

Modification functions

If you choose to modify the model files you must not change the files `reactions`, `metabolites`, `equilibriums`, `TR-excluded`, `fluxmin-excluded`, `concentration-ranges-classes` but the files with the extension `.original` instead. After the modification you call `source fasimu`. You may change the file `simulations` any time you wish. The changes come into effect the next time you call `simulate` or `simulate-single`. For other files you have to call update functions as follows:

Modified file	possible	Function for the safe integration of the modification
reactions	no	change reactions.original instead
reactions.original	yes	fasimu-main
metabolites	no	change metabolites.original instead
metabolites.original	yes	fasimu-main
equilibriums	no	change equilibriums.original instead
equilibriums.original	yes	fasimu-main
TR-excluded	no	change TR-excluded.original instead
TR-excluded.original	yes	fasimu-main
fluxmin-excluded	no	change fluxmin-excluded.original instead
fluxmin-excluded.original	yes	fasimu-main
concentration-ranges-classes	no	change concentration-ranges-classes.original instead
concentration-ranges-classes.original	yes	fasimu-main
enzymes	yes	update-enzymes
fluxmin-weights	yes	update-enzymes (only if enzymes are used)
fluxfix	yes	update-fluxfix
fluxbounds	yes	update-fluxbounds
setfluxes	yes	update-fluxbounds
concentration-class-ranges	yes	update-concentration-ranges
simulations	yes	not necessary
expressions	yes	not necessary
cplex-tail.in	yes	not necessary
lp_solve.par	yes	not necessary
lindo.par	yes	not necessary
$glpk_opts	yes	not necessary
targetfluxes	no	not to be used in a FASIMU session
fluxconstraints	no	not to be used in a FASIMU session

Chapter 4

The steps in a FASIMU session

4.1 Starting FASIMU

As mentioned fasimu is invoked by

```
source fasimu
```

There are some options to this call which are indicated by the following identifiers which follow the word fasimu in the command line.

`cplex`, `cplex9`, `cplex10`, `lp_solve`, `glpk` selects the specific solver. This is necessary if the automatic selection of the solver fails or selects not the desired solver. The automatic recognition of the version of cplex calls `cplex` but that might fail if this is a single license installation and another user is using it at the moment. In this case `cplex9` is used for versions up to 9 and `cplex10` for version from 10 on.

`names_in_allout`. Normally the identifiers of the metabolites are written in the file allout.txt which also. If you prefer the names instead, select this option.

`nomodelprepare` Here, only the FASIMU functions are defined but any action on the model is omitted. This is useful if only a file allout.txt or evaluation.txt from a previous run shall be analyzed or used with the FASIMU functions, for instance for the visualization.

`debug` Some debug messages in the course of the fasimu invocation.

4.2 The control file `simulations`

The general structure is as follows:

- Each line describes a single simulation
- Each line contains four tab-separated fields: name, objective, constraint, evaluator, comment. Each of the fields may contain several tokens, separated by the space character.

First column: Name

primary key, must be a unique, simulations are identified by its name. may contain space characters

Second field: Objective(s), Targetflux(es)

space separated tokens. A token is either a reaction-ID, a metabolite-ID, or a decompartimentalized metabolite-ID (in this case it is defaulted to the compartment indicated by the variable `default_compartment` (which is ext by default), the default for outside the system). Each token can be

preceded by a real valued coefficient ("1" is omitted, this makes sense only if multiple tokens are used to relatively quantify the target fluxes).Tokens in this field are used without qualifiers (see third column), i.e. targets are always defined as reactions working in forward direction or metabolite export across the system boundary.

The following keywords can also be used:

Keyword	Description
max	The following tokens represent fluxes/metablites to be maximized
min	The following tokens represent fluxes/metablites to be minimized
moma	To be used if `$optimization_call` is `compute-moma` or `compute-room` ...
shlomi	To be used if `$optimization_call` is `compute-shlomi` ...

Third column: System boundary tokens

Simplified syntax

Each token is headed by `-`, `+`, `=`, or `%`, followed by an identifier as above.

	Reaction	Comment	Metabolite	Comment
+	forward direction	definition of forward/ backward direction: see below	product	export of this metabolite across the system boundary is allowed
-	backward direction	definition of forward/ backward direction: see below	substrate	import of this metabolite across the system boundary is allowed
=	not applicable	allowing non-zero flux through reactions is the default	product or substrate	import or export across the system boundary are allowed
%	forbidden	flux through reactions must be zero	not applicable	zero flux through exchange reaction across the system boundary is the default

The rules dictated by the above constraints are combined with flux bounds given in the file `reactions`.

Explicit boundary syntax

Another syntax is possible in the constraints section:

Syntax	Comment
`<identifier> = <number>`	Fixed flux for the respective reaction or system exchange
`<identifier> <= <number>`	Upper bound for the respective reaction or system exchange
`<number> <= <identifier>`	Lower bound for the respective reaction or system exchange
`<identifier> >= <number>`	Lower bound for the respective reaction or system exchange
`<number> >= <identifier>`	Upper bound for the respective reaction or system exchange
`<number> <= <identifier> <= <number>`	Range for the respective reaction or system exchange

There must a space characters surrounding the `<=` or = character(s). Instead of `<=` also `<` can be written but the result is identical because in the optimization software (and in fact for the underlying theory of optimization) strictly "less than" is not implemented.

File content syntax

In this column file-content-replacement may avoid repeating long lists several times: When the name of a text file appears in this column, its name is replaced by the file content.

```
.. stddef ...
```

where the file stddef.txt contains the lines

```
Glc_ext
0 <= PFK <= 2
+CO2_ext
```

is equivalent to the constraint definition

```
.. -Glc_ext 0 <= PFK <= 2 +CO2_ext ...
```

Precendence/Overwrite

If there is more than one token constraining the same reaction, only the rightmost constraint is valid, the constraints further to the left are ignored. In other words, the list of constraints is evaluated from left to right and the constraints regarding the same reaction are overwritten. Note that they are not combined: the constraints

```
-Glc_ext ... -8 <= Glc_ext <= 8
```

are interpreted as the glucose exchange flux between (-8,8), not (-8,0).

Fourth column: evaluator

This is used to ensure a quick overview whether a simulation is successful (indicated by a + sign in the second column of `evaluation.txt`). It can be either zero (this means this simulation is intended to fail), one (the existence of a solution suffices), or one (or several) token(s) as above in which case it is checked whether the reaction associated with this token carries a nonzero flux in the flux solution. A metabolite ID can be

- its model identifier with or without compartment
- metabolite name with or without compartment

If more than one evaluator is given the simulation is considered to be satisfied if all evaluators are satisfied. However, the comment in column 3 of `evaluation.txt` indicates which of the tokens are tested successfully.

Fifth column: comment

This is technically not used in FASIMU but it is a good idea to describe the simulation semantically here.

4.3 Controlling the computation function

There is one function for all sorts of flux optimization called `compute-FBA`. The variable `$optimization_call` should be set to one of the functions below together with parameters, e.g. `optimization_call=compute-FBA -T A -F e -s 1 -w 0.0001''`.

The flux minimization: compute-FBA/compute-FBA -F

A flux distribution obeying the flux-balance condition is computed. It minimized the fluxes as a general rule, but there are some variants, controlled by the single parameter. The default is an implementation of [5] where the forward fluxes have the weight 1 and the backward fluxes have the weight equal to the equilibrium constant (given in the file `equilibriums`). If the parameter `-F s` is given the weights of the forward and backwards flux are divided by $\sqrt{1+\mathrm{K_{eq}}^2}$, according to [7]. The parameter setting `-F 1` follows the same idea, both weights are divided by $1+\mathrm{K_{eq}}$. For the parameter setting `-F 2`, the forward flux has the weight $1/1+\mathrm{K_{eq}}^2$, the backward flux the weight $\mathrm{K_{eq}}^2/1+\mathrm{K_{eq}}^2$. For the parameter `-F 0`, the backward flux has the same weight as the forward flux, regardless of the value in equilibriums. This is equivalent to the case where no equilibrium data is given at all. For the two options `-F e` and `-F E` the file enzymes must be available. Here, weights are not given for the fluxes themselves but for the enzyme with respect to a reaction. The general principle is also given in [6]. The difference between e and E is that for e both directions of the

reaction receive the same weight where for E the backward flux is multiplied with equilibrium constant, similar to the default.

Allowing restricted fitness: compute-FBA -f

This implements the generalized flux minimization principle [6]. Fluxes must not strictly obey the given objective (in FASIMU, the objective tokens in `simulations`) but are allowed to deviate. Thus, the optimization maximizes the fitness as the first priority and then minimizes the fluxes (as above) as the secondary goal.

The fitness function ranges from zero to one, where one represents maximal fitness. This figure is printed in the output files.

The option `-f` takes 2 parameters. The first one is the type of the fitness function, ranging from 0 to 7. Default is 0. For solvers LINDO, lp_solve and GLPK quadratic scoring functions are not implemented, so the type may only range from 3 to 7, and the default is 5. The types are distinguished by the way the aberration to the target flux is combined: by the Euklidian norm (0,1,2), by the 1-norm (3,4,5), by the maximum norm (6,7). The are also distinguished whether the distances are normalized or not, i.e. if absolute or relative distances are considered. Type 1 is special as it involves a partial normalization. Type 3 is a very easy and fast implementation but for this it is necessary that the flux values have the same sign as the target value for the target fluxes (it is guaranteed in the computation).

Type	Norm	Adjustment	Fitness score	Restriction
0	Euklidian	none	$1-\sqrt{\frac{\sum \Delta v_i{}^2}{\sum L_i{}^2}}$	
1	Euklidian	partly (linear)	$1-\sqrt{\frac{\sum \frac{\Delta v_i{}^2}{\lvert L_i \rvert}}{\sum \lvert L_i \rvert}}$	
2	Euklidian	full	$1-\sqrt{\frac{1}{n}\sum \frac{\Delta v_i{}^2}{L_i{}^2}}$	
3	linear	full	$1-\frac{1}{n}\sum \frac{\lvert \Delta v_i \rvert}{\lvert L_i \rvert}$	$\text{sgn}(v_i)=\text{sgn}(L_i)$
4	linear	none	$1-\frac{\sum \lvert \Delta v_i \rvert}{\sum \lvert L_i \rvert}$	
5	linear	full	$1-\frac{1}{n}\sum \frac{\lvert \Delta v_i \rvert}{\lvert L_i \rvert}$	
6	maximum	none	$1-\frac{\max\{\lvert \Delta v_i \rvert\}}{\max\{\lvert L_i \rvert\}}$	
7	maximum	full	$1-\max\left\{\frac{\lvert \Delta v_i \rvert}{\lvert L_i \rvert}\right\}$	

L_i is the target flux value. $\Delta v_i = v_i - L_i$ is the distance of the actual flux value to the target flux value. The sums and sets are over the target reactions i.

The second parameter is the weight with respect to the weight of the fluxes. Technically, the optimizer does not supply a hierarchic optimization, thus, a large weight has to be given to the fitness score. The default is 10^6.

Fitness maximization is closely related to MOMA (minimal metabolic adjustment) and can in fact implemented by defining the targetfluxes according to a reference solution. However there is also a special FABASE function `compute-moma` for this purpose.

Minimizing a selected target: `compute-FBA -m`

This function allows to minimize one or more reaction fluxes, usually applied to the inward transport process of substrates. The parameter to this option is one or more reaction identifiers.

There are two ways to use this option in FASIMU. In the first one `-m` is included in `$optimization_call`. In the `simulations` file the objective (second column) is left blank (or used to fix some other fluxes). The disadvantage of this way is that for all simulations the minimization of the same reaction/metabolite is performed.

The second, strongly recommended, way does not have this restriction. The variable `$optimization_call` contains a compute-FBA call without the -m option but possibly with other pa-

rameters. In the simulations file the objective (second column) is min <rea/met> ... with one or more tokens (reaction identifer, metabolite identifier or names). The subroutine of simulate executing this simulation automatically adds -m <reaction-ID> to $optimization_call. Obviously, this only works if the $optimization_call is compute-FBA with possible further parameters, but not other calls as compute-shlomi (which wouldn't make much sense anyway).

Maximizing a selected target: compute-FBA -b

This option allows to maximize one or more reaction fluxes, usually applied to the biomass synthesis process or an important product [2]. The parameters to this function are the reaction identifiers.

Here the same mechanisms can be used as in the previous section. The respective keyword is max instead of min.

Thermodynamic feasibility computations: compute-FBA -T

This option switches on the criterion that a positive flux through a reaction must be accompanied by a negative Gibb's free energy the latter being dependent on standard Gibb's energies and ranges of allowable concentrations. The so-called criterion of thermodynamic realizability (TR) is described in detail in [8]. This option requires equilibrium and concentration ranges to be set (see above).

TR is only a constraint, the flux objective is set by the above options -F, -m, and -b, with the default of -F 0 if just called with compute-FBA -T.

The TR computation not only predicts fluxes but also metabolite concentrations, stored in the file concentrations.txt. With the option -d given to compute-FBA a more comprehensive report on concentrations is stored in the file TR-FBA-concentration-doc.txt comprising also the applied ranges. -d gives also further information files: TR-FBA-potential-doc.txt, TR-FBA-potential-doc2.txt, and TR-FBA-summary.txt. Potentials (see below) are the sums of logharithms of the reactants plus the logharithm of the equilibrium constant. The documentaries on potentials show it for individual reactions. The summary shows the whole model togehter with the flux distribution and the concentrations.

For good concentration predictions reasonable soft bounds (switched on with the -s [<weight>] option) and set points (switched on with -w [<weight>]) should be supplied for the concentrations. Typical call is "compute-FBA -T -s 1 -w 0000.1". The default weight for the soft bounds is 1, the default weight for the setpoints is 10^-5, so this call is equivalent to compute-FBA -T -s -w. Note that it is not enough that the soft bounds and the setpoints are defined in concetration-class-ranges, they are only used if -s or -w, respectively, are given.

For the TR criterion a type identifier can be given which controls the way, zero fluxes and thermodynamic potentials are treated. Basically, a non-zero flux and a non-zero potential must have the same sign. (Thermodynamic potential here is the negative Gibb's energy change divided by RT.) But it is not clearly defined if one of the values is zero. There are two ways: in the relaxed form (a or A) a zero potential is compatible with any flux value, and a zero flux value is compatible with any potential. In the strict form (b or B) a zero potential is only compatible with a zero potential and vice-versa. The differences in the biochemical interpretation will not be covered here. But from the computational side: types b and B are much harder to compute (on more binary variable for each reaction) and the system is much more likely to be infeasible. The uppercase letters refer to an implementation of TR with conditional clauses which is more robust, numerically stable, and accurate in the computation but is only available in the solver CPLEX version 10 and higher. The lowercase letters refer to the so-called bigM implementation which is the standard for other solvers.

A trailing "r" in the type identifier changes the way reaction marked as irreversible are dealt with. By default, there is still the binary variable. For a zero flux, the potential can have either sign. By the setting "r" there is no conditional variable but the sign of the potential is fixed to the sign of the irreversibility constraint. If the flux is non-zero, that is not a difference. However, for a zero flux, the constraint on the potential is still active, where in the default way, there is also a potential with the opposite sign possible.

Again, the biochemical implication will not be discussed here. From the computational side, the setting "`r`" accelerates the computation but also increases the likelihood that the system is unfeasible.

Type	Zero flux	Zero potential	Irreversible reaction	comment
A	q arbitrary	any flux		Conditional clause implementation
B	$q = 0$	$v = 0$		Conditional clause implementation
a	q arbitrary	any flux		bigM implementation
b	$-\epsilon < q < \epsilon$	$-\epsilon < v < \epsilon$		bigM implementation
Ar	q arbitrary	any flux	potential sign fixed	Conditional clause implementation
Br	$q = 0$	$v = 0$	potential sign fixed	Conditional clause implementation
ar	q arbitrary	any flux	potential sign fixed	bigM implementation
br	$-\epsilon < q < \epsilon$	$-\epsilon < v < \epsilon$	potential sign fixed	bigM implementation

See the section about concentration ranges above as they are necessary for the TR computation.

Further options of `compute-FBA`

There is another parameter worth to mention: `-t <seconds>` which restricts the computation time of a single optimization.

MOMA: `compute-moma`

This function implements MOMA [16] using the fitness function (see above). The original paper uses unnormalized Euklidian distance but that can be changed by setting the `-f` option. In fact, all options of compute-FBA can also be given to compute-moma. The Euklidian distance can only be used if the solver is CPLEX, otherwise it is switched to the non-normalized linear type (-f 5). `compute-moma` has its own help text available with `compute-moma -h`.

ROOM: `compute-room`

This function implements Regulatory on/off minimization [17]. As opposed to compute-moma it is a separate implementation and has only a few parameters to be displayed with `compute-room -h`. It is possible to change the relative and absolute threshold (δ and ϵ in the original paper are set by -d and -e respectively).

Expression-based flux prediction: `compute-shlomi`

The function `compute-shlomi` implements the algorithm to predict the active subnetwork depending on a expression profile (which can be a protein or transcript profile) [18]. The profile is given in the file `expressions`, which is white-space separated. The first token is the reaction identifier, the second is an expression value, which can be a float number, a binary or a three valued measure. Its interpretation is controlled with the threshold values given -l and -u. For compute-shlomi -l 0.4 -u 0.6 transcript values lower than 0.4 are considered as off, higher than 0.6 as on, and values in between are considered as grey zone and are excluded from the algorithm. Reactions not referred to in expressions are also excluded from the algorithm — this is equivalent to the treatment of grey zone values. See also the help available with `compute-shlomi -h`.

Combined expression-based flux prediction: `compute-FBA -x`

Here, the expression-based flux distribution is not the only component of the scoring function as above. It is combined with scoring components of other options (for instance flux minimization). See [9] for details.

The option `-x [<weight>]` switches the algorithm and also gives the relative weight, default weight is 10^3. The same parameters as above are available: `-xu <number>` is the upper threshold on the expression level (default 0.5), `-xl <number>` is the lower threshold on the expression level (default 0.49), `-xs <number>` sets the significant flux threshold (default 1).

4.4 Perform simulations: `simulate`

The function call `simulate` performs all simulations described in `simulations` and stores the complete result in `allout.txt` and a short overview in `evaluation.txt`. To just compute a single simulation identified by its name call `simulate-single "<name>"` where the quotes can be omitted if the name contains no space characters. To store the solutions requires considerably disk space for large networks, `simulate-noallout` performs the computations but suppresses to write the solutions.

The call of `simulate` deletes the files `allout.txt` and `evaluation.txt` as the first step, thus, it is guaranteed that after a simulate call those files contain the information on one complete set of simulations. The call `simulate-single` however appends the result to `allout.txt` and `evaluation.txt`. This way they may contain several solutions of the same simulation.

The call `compute-FBA` stores information which is overwritten by the next call which is the case if `simulate` is called and there is more than one simulation. If you are interested in this information, call `simulate-single` instead and save the information in separate files. See the following table for the information:

**Overwrittem information**	
`problem.lp`	complete optimization problem description
`solver.out`	solver output
`variables.out`	values of all variables
**Appended information**	
`evaluation.txt`	summaries
`allout.txt`	solutions
`cplex-times`	Running times (CPLEX only)

4.5 The evaluation file: `evaluation.txt`

This file is tab separated:

1. Name of the simulation
2. + or − depending if the computed result is in accord with the expectation
3. A comment on the evalution

There are several bash functions which provide convenient views on the result:

`negeval` gives only lines in evaluation.txt which have not the desired result.

`inacceval` scans the file for warnings on the inaccuracy of results (switched on by the `-c` option of `compute-FBA`).

4.6 The comprenhensive solution file: `allout.txt`

The file contains all information on the solutions: flux values, concentration values if applicable in a human readable form. Each solution begins with a line with many `\#` characters and the name of the simulation. Then a message on computation and possible warnings follow. After that the reaction fluxes follow in a tab separated format, the first item is the reaction identifier, the second item is the flux rate, the third is the equilibrium constant used, the fourth is the reaction equation, the fifth are possible annotations of the reactions (given in reaction-names). The reactions are normally written with the identifier of the metabolites. However, it can also be written using the names of the identifier by invoking fasimu with:

```
source fasimu names_in_allout
```

Zero fluxes are omitted throughout, although occasionally fluxes with flux rates close to zero may appear in large networks which are numerically difficult. The first fluxes denoted in the solution are the fluxes across the systems boundary (which are not fluxes in the metabolic reaction systems), identifiable by the trailing "`_tr`" in the reaction identifier and the trailing "`_ex`" in the metabolite identifier in the reaction equation. Then the regular fluxes follow. If applicable, i.e. if `compute-FBA -T` was used, concentrations of the metabolites involved in the solution follow, in a tab-separated format: the first item is the metabolite identifier, the second is the concentration value, and the third are the concentration ranges used in the computations which might include soft bounds and set points if they have been used.

4.7 Prepare files for the visualization

FASIMU itself has no visualization capabilities on its own, however it includes plugins for the easy integration of computed flux modes in other packages.

Visualization in Cytoscape

For the visualization in [11] in combination with the flux analysis plugin, the call of `allout2valfiles` transforms the solutions recorded in allout.txt in separate files in a directory `val`.

Visualization in BiNA

For the visualization in BiNA [13] in combination with the flux analysis plugin (`http:/binafluxmodel.sourceforge.net/`), the call of `allout2bina` transforms the solutions recorded in allout.txt in separate files in a directory BiNA. The names of the files are the respective simulation names. There are two parameters to this function. The metabolites (without a compartment identifier) following "`-a`" are defined to be alias metabolites (see BiNA documentation), metabolites which are not drawn as one node in the graph but as many nodes possible distributed throughout the graph. E.g.

```
allout2bina -a ATP ADP NADH NAD
```

defines these metabolites to be aliased in all occurrences. BiNA is capable to define alias function for each reaction separately but this has to be modified manually, see the description of the BiNA-flux analysis file format. The parameter -c defines RGB color values for the color of metabolite nodes depending on the compartment. The syntax is as follows: ¡compartment¿[¡numR¿,¡numG¿,¡numB¿]. This sequence must be written without space characters. The respective values range from 0 to 255. In the -c option an arbitrary number of such items may be given. Example:

```
allout2bina -c cyto[245,45,226] mito[66,212,244]
```

Note that if all three numbers are low the color is very dark and the black letters of the metabolite name may not be readable.

A previous directory BiNA will be removed by this function. If the file `allout.txt` contains more than one flux distribution with the same name (this happens if the same simulation is run over again with simulate-single), a underscore character and a number (starting from 0) is appended to the name of the simulation to avoid overwriting.

Visualization in CellNetDesigner

For the visualization in [12] , the files stored in the `val` directory by `allout2valfiles` can also be directly be used for the visualization in CellNetDesigner by the ReadMode function. Of course, CellNetDesigner requires a ready-made image of the network.

Chapter 5

Further functions in FASIMU

5.1 Prune to *ad functionem* networks: `prune-network`

The function checks for each reaction whether a positive or negative flux is possible. This depends on the selection of the optimization protocol (variable `$optimization_call`) and the contents of the file `stdexch.txt` which defines the system boundary. See [4] for details on the method. Both, the exchange metabolites and the functional metabolites mentioned in the article must be included in the same file `stdexch.txt`.

The result of the function is a new network in the subdirectory `sub`. The blocked reactions are removed from the network. If pnly one direction is blocked it is marked as irreversible in the newly created file `sub/reactions`. The resulting files are a valid FABASE model, i.e. it does not contain the FASIMU pseudo reactions controlling the systems boundary.

If an error message "`bash: declare: -A: invalid option`" is shown, you should update your bash to a version higher than 4.

If that is not possible you can use the function `prune-network-simple` which should obtain the same result but is much slower as doesn't use already computed flux solutions to reduce the number of required optimization objectives.

5.2 Flux-variability analysis

The function `make-FVA-simulationsfile` prints a simulations file applicable to perform a flux-variability analysis [14,15] which should be directed to `simulations`. There are two other elements to be taken care for a meaningful FVA. The first is the file with the predefined name `stdexch.txt` which defines the constraints valid in all defined simulations and it should be used to allow input of substrates and output of waste products and metabolic objectives. The second is the definition of flux boundaries. If there would be no flux restrictions probably many of the fluxes were unbounded in which case the FVA would be meaningless. It is recommended to either restrict all input fluxes or all output fluxes.

The function `FVA-valfiles-chart` interprets the result files of a FVA to produce a chart, list of reactions with their respective minimum and maximum. It uses the files in the directory `val` thus, it is necessary to call `allout2valfiles` beforehand. See tutorial for the normal work flow:

```
make-FVA-simulationsfile > simulations
allout2valfiles
FVA-valfiles-chart
```

This version of FVA directly maximizes and minimizes the problem. If there are problems that the optimization problem is not bounded, you can use another form of FVA which only checks whether it is possible to find a flux solution with a unit flux in both directions for each reaction:

```
make-unity-FVA-simulationsfile > simulations
allout2valfiles
FVA-valfiles-chart
```

5.3 Check the essentiality for a given set of simulations: `check-essentiality`

This functions checks the essentiality of reactions with respect to the defined simulations. First, the actual simulations (defined by the `simulations` file and the variable `optimization_call`) are performed. Based on the results a new set of simulations is defined implementing a single knock-out for each reaction contained in the solution of the respective. Note that this is much more efficient than to check every reaction — a reaction can only be essential if it appears in a reference solution.

The result is written concisely in essentiality-report.txt. Each line refers to a simulation in the original set. The lines are tab-separated, the first token is the identifier of the simulation and the second is either

- the comment `failed` if the original simulation failed
- the comment `no essentials` if they are no essential reactions, or
- `essentials`: followed by a space separated list of reaction identifiers referring to the essential reactions.

Other intermediate files can also be inspected:

- `evaluation.txt` contains the result of the reference run,
- `simulations_essentiality` contains the newly defined simulations,
- `evaluation_essentiality.txt` contains their results.

5.4 Functions creating common `simulations` files

The following functions produce simple `simulations` files for common applications.

Check the producability of all metabolites

```
make-fullproducibility-simulationsfile > simulations
```

This `simulations` file requires that the system boundaries are stored in the file `stdexch.txt`.

Check the degradibility of all metabolites

```
make-fulldegradibility-simulationsfile > simulations
```

This `simulations` file requires that the system boundaries are stored in the file `stdexch.txt`.

Check the network on leaks

The following `simulations` file makes two checks:

- Can any metabolite be produced without any inward transport while any outward transport is allowed?
- Can any metabolite be degraded without any outward transport while any inward transport is allowed?

This check is very similar to the check proposed by [3].

```
make-leakcheck-simulationsfile > simulations
```

The function also writes two additional files, `any_outwards.txt` and `any_inwards.txt`, from all metabolites.

Exclude that metabolites can be created without substrates

```
make-futileproducibility-simulationsfile > simulations
```

This `simulations` file requires that the excretable products stored in the file `stdwaste.txt`.

This is a weaker test as the leak test in the previous section since only some given waste products are allowed as excretion products and not any metabolite. It might still be a worthwhile check if the results of the former are difficult to interpret.

Exclude that metabolites can be degraded without excretion products

```
make-wastability-simulationsfile > simulations
```

This `simulations` file requires that all possible substrates are stored in the file `stdsubstr.txt`.

5.5 Functions creating common exchange files referenced in a `simulations` file

Access the system boundaries in the original FABASE model

```
make-standard-exchangables > stdexch.txt
```

This functions writes the metabolite marked as "open boundary" in the orignal FABASE model with the qualifier "=", meaning it can be exchanged in both directions. If this model was created with `sbml2fa` it is exactly the boundary condition of the original SBML file.

```
make-standard-wastables > stdwaste.txt
```

does the same but adds the qualifier "+" meaning that these substances can be excreted to be used by a `simulations` file created by `make-futileproducibility-simulationsfile`.

```
make-standard-substrates > stdsubstr.txt
```

does the same but adds the qualifier "-" meaning that these substances can be imported to be used by a `simulations` file created by `make-wastability-simulationsfile`.

5.6 Network printing functions

The present FASIMU or FASBASE network can be converted in other formats. Some of these functions have already been executed and their results are stored in the current directory:

**File**	**format**	**generating function**
`reactions.txt`	plain text format	`reactions-printout equilibriums`
`reactionsa.txt`	dito, stoichiometrix factor 1 omitted	`reactions-printout equilibriums condensed reanames`
`reaclear.txt`	text format, metabolite names instead of identifiers	`reactions-printout equilibriums cleartext`
`reacleara.txt`	dito, stoichiometrix factor 1 omitted	`reactions-printout equilibriums condensed cleartext reanames`
`network.sbml`	SBML version 2 level 4	`print-sbml`

All functions print their output to the screen. They can be used both in a FASIMU or FABASE session. The do not print the pseudo reactions which FASIMU adds.

Plain text format: `reactions-printout`

This functions writes plain text format of the recent network. It is a tab-separated file. The first column contains the reaction identifer, the second column the reaction equation, separated by spaces. It is written to standard output, to save it to a file, call

```
reactions-printout > network.txt
```

More columns are possible added by the options as follows.

cleartext Write metabolite name instead of identifers.

condensed Suppress the stoichiometric coefficient.

equilibriums Write the equilibrium constant in the second column.

reanames Add a last column with the respective content of `reaction-names`.

SBML format: `print-sbml`

This functions writes a SBML version 2, level 4 to the standard output, to save it to a file use:

```
print-sbml > network.sbml
```

Options:

noconcentrations Normally the concentrations stored in `concentation-class-ranges` in conjunction with `concentration-ranges-classes` are stored in a `<listOfConstraints>` section. The soft bounds result in a warning if exceeded, whereas the hard bound cause an error message. The set points values are written as the inital concentration variable in the species tag. This options suppresses this altogether.

nospeciestype Normally species are assigned a speciesType, the metabolite identifier and name without the compartment identifier. This options suppresses this.

noequilibriums Normally the equilibrium constants are stored in a tag: `<parameter id="equilibrium_constant" value=".."/>` inside a `<listOfParameters>` in a `<kineticLaw>` section for every reaction. This options suppresses this.

METATOOL format: `print-metatool-network`

Simple function to transform the current network into METATOOL format, simlar to the above functions. It has no parameters.

expa format: `print-expa-network`

Simple function to transform the current network into expa format, simlar to the above functions. It has no parameters.

Chapter 6

Using FABASE without FASIMU

The question arises why the two layer structure FABASE vs. FASIMU which resulted from historical reasons is still retained. The answer is that it may still be worthwhile to use FABASE on its own so this possibility is still supported.

To use FABASE without FASIMU is a reasonable alternative if FASIMU's simulations concept is not needed, i.e. if the focus is on a single optimization problem (biomass maximization for instance). Reasons might also be related to computation times: FASIMU increases the number of reactions and metabolites considerably (one extra reaction and metabolite for each metabolite) to control the systems boundaries. The intrinsic problem complexity is not changed by this modification, however, the files get larger, and some scripts may be affected by this.

The main reason however to use FABASE alone is for experimentation. In a typical FASIMU session, the basic information files (the LPF-files) are overwritten frequently and some flow of information is not visible. It may even be sensible to modify certain LPF-files in a text editor to obtained algorithmic features not yet (or never be) implemented in FASIMU. Also a combined approach is possible: start with FASIMU (including the added system boundary control) and continue with FABASE functions.

Technically, the main difference between a FASIMU and FABASE session is that in a FABASE session you call `compute-FBA` (or other `compute-` functions) directly from the command line where in a FASIMU session you put the respective `compute-` functions into the variable `$optimization_call` and enter `simulate` or `simulate_single` in the command line — the `compute-` functions are called indirectly. The other important difference is that in a FABASE session you have to put the objectives in the file `targetfluxes` and the constraints in the file `fluxconstraints` where in a FASIMU session the objectives and constraints are condensed in a single line of `simulations` and `targetfluxes` and `fluxconstraints` are written upon this information. Simply put: FABASE: more control but also more manual work. The startup of a FABASE section is:

```
source fasbase
```

One example of a small FABASE session is given in the tutorial about *E. coli*: the modification of the equilibrium constants into a set obeying the WEGSCHEIDER condition. Here, the function `well-formed-equilibriums` is called directly on the model.

6.1 Computation functions

The principal optimization function: `compute-FBA`

The more comprehensive description can be found in section 4.3. Here, a more technical description can be found. The basic usage is:

```
compute-FBA [options]
```

`compute-FBA` on its own is translated to `compute-FBA -F`. Description of the options follow.

Options for flux minimization: `-F` [`<fluxmin-type>`]

**Type**	**weight of forward flux**	**weight of backward flux**
h	1	K_{eq}
0	1	1
1	$\frac{1}{1+K_{eq}}$	$\frac{K_{eq}}{1+K_{eq}}$
2	$\frac{1}{1+K_{eq}^2}$	$\frac{K_{eq}}{1+K_{eq}^2}$
s	$\frac{1}{\sqrt{1+K_{eq}^2}}$	$\frac{K_{eq}}{\sqrt{1+K_{eq}^2}}$
e	<enzyme cost>	<enzyme cost>
e	<enzyme cost>	<enzyme cost> K_{eq}

The type can be omitted in which case it is `0` if `-T` is also set, and `h` otherwise.

Specific minimization: `--m` [`<rea-ID>`] ...

The flux of the given reaction(s) is minimized. For this to be useful it is necessary that at least one flux is set to a nonzero value (e.g. with the `setfluxes` file), otherwise the zero solution will be computed.

Specific maximization: `--b` [`<rea-ID>`] ...

The flux of the given reaction(s) is maximized. For this to be useful it is necessary that wither the intake or the output fluxes are restricted (e.g. with the `fluxbounds` file), otherwise the problem becomes unbounded.

Fitness maximization: `--f` [`<type>` [`<weight>`]]

Type	Norm	Adjustment	Fitness score	Restriction
0	Euklidian	none	$1 - \sqrt{\frac{\sum \Delta v_i{}^2}{\sum L_i{}^2}}$	
1	Euklidian	partly (linear)	$1 - \sqrt{\frac{\sum \frac{\Delta v_i{}^2}{\lvert L_i\rvert}}{\sum \lvert L_i\rvert}}$	
2	Euklidian	full	$1 - \sqrt{\frac{1}{n}\sum \frac{\Delta v_i{}^2}{L_i{}^2}}$	
3	linear	full	$1 - \frac{1}{n}\sum \frac{\lvert\Delta v_i\rvert}{\lvert L_i\rvert}$	$\mathrm{sgn}(v_i) = \mathrm{sgn}(L_i)$
4	linear	none	$1 - \frac{\sum \lvert\Delta v_i\rvert}{\sum \lvert L_i\rvert}$	
5	linear	full	$1 - \frac{1}{n}\sum \frac{\lvert\Delta v_i\rvert}{\lvert L_i\rvert}$	
6	maximum	none	$1 - \frac{\max\{\lvert\Delta v_i\rvert\}}{\max\{\lvert L_i\rvert\}}$	
7	maximum	full	$1 - \max\left\{\frac{\lvert\Delta v_i\rvert}{\lvert L_i\rvert}\right\}$	

The `weight` is multiplied with the above coefficient. Fitness maximization can be combined with flux minimzation and specific minimization but not with maximization.

Using expression profiles

This function requires the file `expressions` to be present, giving the expression values. The call is:

```
compute-FBA -x [<weight>] [-xu <upper>] [-xl <lower>] [-xs <significant>]
```

The option `-x` switches the function on, meaning that specific terms are added to the scoring function. Thus, this feature can be combined with other optimizations. Defaults are

Parameter	meaning	default value
<weight>	relative weight with respect to scoring function	1000
<upper>	upper threshold on positive expression	0.5
<lower>	lower threshold on negative expression	0.49
<significant>	threshold on the significance of the absolute value of a flux	1

Thermodynamic realizability

Options for TR:

Option	purpose	default value
-T	use TR criterion, default type	
-T [[A-Ea-e]r?]	use TR with type identifier	A
-s [<weight>]	use soft bounds	1
-w [<weight>]	use set points	10^{-5}
-k <number>	potential tolerance maximum	
-K <number>	penalty multiplicator for potential tolerance	
<num>..<num>	alternative way to enter concentration values	

TR types are summarized in this table:

Type	Zero flux	Zero potential	Irreversible reaction	comment
A	q arbitrary	any flux		Conditional clause implementation
B	$q = 0$	$v = 0$		Conditional clause implementation
a	q arbitrary	any flux		bigM implementation
b	$-\epsilon < q < \epsilon$	$-\epsilon < v < \epsilon$		bigM implementation
Ar	q arbitrary	any flux	potential sign fixed	Conditional clause implementation
Br	$q = 0$	$v = 0$	potential sign fixed	Conditional clause implementation
ar	q arbitrary	any flux	potential sign fixed	bigM implementation
br	$-\epsilon < q < \epsilon$	$-\epsilon < v < \epsilon$	potential sign fixed	bigM implementation

Program control options

Option	purpose
-p	prepare only 'problem.lp', do not compute
-t <seconds>	timeout in seconds, finish (single flux computation), default 300, 0 means unlimited time
-n <nodes>	abolish computation number of nodes, default 5000, 0 means unlimited number of nodes (CPLEX only)
-e <1..4>	MIP emphasis setting to be tried out in critcal size models (CPLEX only)
-C "<param-line>"	Give this line to CPLEX
-c	check the results with another cplex run
-d	print documentation files for thermodynamic feasibility

6.2 Further functions in FABASE

Ensure standard directions

The function `ensure-standard-directions` ensures the convention that the reactions are written such that the standard Gibb's free energy is negative, i.e. the equilibrium constant is larger or equal to 1. In other words, if all metabolites have a concentration of 1M each reaction proceeds in forward direction. This condition is assumed in the formulation of the flux minimization algorithm [5], which is implemented as `compute-FBA -F h` in FASIMU. The function simply exchanges products and substrates in the reaction equation for the respective reactions, also modifies the boundaries stored in the file `reactions` accordingly,

and replaces the equilibrium constant by its inverse. It does currently not change the contents of `setfluxes`, `fluxconstraints`, `fluxbounds`, `fluxfix` etc. . The user has to take of that.

The function modifies the files `reactions` and `equilibriums`. However, it does not regenerate all information depending on these files. Therefore, it does not work just to continue with the FASIMU/FABASE session. The recommended way to use the function is to create a new model with the modified files `reactions` and `equilibriums:`, as follows, assuming the original model is in `$modeldir/model1`:

```
mkdir newdir
cd newdir
cp -R $modeldir/model1/*
source fabase
ensure-standard-directions
cp -R $modeldir/model1 $modeldir/model2
cp reactions equilibriums $modeldir/model2
rm *
cd ..
rmdir newdir
```

Still, it is possible to restart a FABASE session with modified `reactions` file, just call `fabase-main`. Generally, it is not a good idea to modify the file reactions in a FASIMU session, since fasimu modifies the model, but it is possible with:

```
ensure-standard-directions
rm reactions.original equilibriums.original
fasimu-main
```

Well-formed equilibriums

For thermodynamic computations it is favorable if the standard reaction free energies are compatible to each other, in other words, they obey the WEGSCHEIDER condition. Experimental values from different sources obviously do not exactly comply with this rule. Values derived from formation energies by prediction methods [10] should do so but this is not always the case due to computational errors. The function `well-formed-equilibriums` attempts to force this condition with changes to the data given in `equilibriums`. It is done with an error minimization which is either linear (-l option) or quadratic (-q only available for solver CPLEX). The latter usually spreads the modification values on more reactions. This algorithm is described in [8]. There are two files modifying the algorithm: If a reaction is included in the file `trusted-equilibriums` it will not be modified. If a reaction is included in the file `untrusted-equilibriums` it will primarily be changed. The corrected set of equilibrium constants will be written to `corrected-equilibriums` and a modification report is written to `eq-corr-report.txt`.

It is recommended not continue the FABASE or FASIMU session with a modified `equilibriums` file but to generate a separate model instead as described in the previous section (Ensure standard directions). However, in a FABASE session, fabase-main can be called, or in FASIMU session fasimu-main can be called to regenerate dependent information.

Another recommended way to modify the model prior to a FASIMU session is to start a small fabase session just for the modification of the model, and then start FASIMU in the normal way:

```
source fabase
well-formed-equilibriums
source fasimu
```

This also works with `restrict-equilibriums` and `ensure-standard-directions`.

6.3 Modify model files in a FABASE session

The recommended way to handle your models

Generally, I do not recommend to modify the files in the directory in which you are working with FABASE. You should keep the original models in separate directories and call `source fabase` in a fresh directory. Say, you keep and modify your model in a directory `~/model` then proceed like that:

```
cd ~
cp -R model model-compute
cd model-compute
source fabase
<do the computations>
<copy the result files to a designated directory>
cd ..
rm -r model-compute
```

Hoever, for experimentation you can change all input files during the session. The program is capable to regenerate intermediate files but that requires to call functions.

Modification functions

See the following table for the modification functions. If you change one of items below the function on the right hand side must be called. `fabase-main` regenerates every intermediate file, so it can be used if several files are changed.

Modified file	possible	Function for the safe integration of the modification
`reactions`	yes	`fabase-main`
`metabolites`	yes	`fabase-main`
`equilibriums`	yes	`fabase-main`
`TR-excluded`	yes	`update-TR-excludes`
`fluxmin-excluded`	yes	`update-enzymes` (only if enzymes are used)
`fluxmin-weights`	yes	`update-enzymes` (only if enzymes are used)
`concentration-ranges-classes`	yes	`update-concentration-ranges`
`concentration-class-ranges`	yes	`update-concentration-ranges`
`fluxbounds`	yes	`update-fluxbounds`
`setfluxes`	yes	`update-fluxbounds`
`fluxconstraints`	yes	`update-fluxbounds`
`targetfluxes`	yes	`make-vset-lpf`
`enzymes`	yes	`update-enzymes`
`fluxfix`	yes	`update-fluxfix`
`expressions`	yes	not necessary
`cplex-tail.in`	yes	not necessary
`lp_solve.par`	yes	not necessary
`lindo.par`	yes	not necessary
`$glpk_opts`	yes	not necessary

Chapter 7

Customization of the external solvers

Large networks in combination with computational expensive constraints such as

- Thermodynamic realizability (requires Boolean variables, thus, computing a flux distribution is now at least a mixed-boolean linear problem, and the number of variables increases: for each metabolite one Boolean and one real-valued variable)
- Set points for concentration values (`-w` option) turn the problem into a quadratic objective problem
- Use of the fitness maximization, types 0...2 also turns the problem into a quadratic constraint problem
- stoichiometric coefficients are real numbers or have a large value (increase the numerical difficulty of the problem, which might cause apparently wrong solutions)

may push the optimization on the brink of their capability. However, all solvers can be customized. Choosing the right parameter switch may put a previously unsolvable problem into reach. You should inspect the file "`solver.out`" which is the output of the solver program stored after each computation call by FASIMU and the instruction manual of the respective solver.

By default, FASIMU instructs the solver to finish a single optimization after 5 minutes. The functions

```
set-timeout <seconds>
```

modifies the configuration of the active solver to change this time span. The value 0 disables the timeout feature.

CPLEX

As FASIMU has been developed with CPLEX, a few default settings have been found to ideally harmonize with difficult FBA optimizations. The parameters are stored in the file `cplex-tail.in` which you can modify to change the values of parameters. You can display them with

```
cat cplex-tail.in
```

The file will not be overwritten by a call of "`source fasimu`"; the changes will be in effect for all further computations in this directory. The defaults can be restored with:

```
restore-defaults-cplex
```

LINDO

Lindo parameters are stored in “lindo.par”. You can display them with

```
cat lindo.par
```

Again, this file will not be overwritten by a subsequent call of “source fasimu”; the defaults can be restored with:

```
restore-defaults-lindo
```

lp_solve

Parameters for lp_solve are stored in lp_solve.par. You can display them with

```
cat lp_solve.par
```

For more information on parameters see http://lpsolve.sourceforge.net/5.5. The file lp_solve.par will not be overwritten by a subsequent call of “source fasimu”; the defaults can be restored with:

```
restore-defaults-lp_solve
```

GLPK

GLPK parameters are stored in the bash variable “$glpk_opts”. The variable contains command line options given to GLPK. Call

```
glpsol -h
```

to see a list of options. To apply different parameters you have to adjust this variable. You can display them with

```
echo $glpk_opts
```

To see a list of possible parameters, call

```
glpsol --help
```

in the command line. The variable is not changed by a subsequent call of “source fasimu”; the default can be restored with:

```
restore-defaults-glpk
```

Chapter 8

Final remarks

8.1 Getting more help

There is also a FASIMU tutorial which may by helpful to you.

Functions help page

Some functions have their own help pages available with the "-h" option: `compute-FBA`, `compute-shlomi`, `well-formed-equilibriums`, `compute-moma`, `compute-room`. FASIMU has an online help page: `fasimu-help`.

Source code

FASIMU is written in the high-level languages bash and gawk so if you are fairly familiar with the syntax of these languages it may be worthwhile to look in the source code. For some functions additional information is available in the comments of the source code. The functions related to FABASE are defined in `/usr/local/bin/fabase`, related to FASIMU in `/usr/local/bin/fasimu` (for the recommended installation places).

FASIMU project website

You might wish inspect the project's website at

http://www.bioinformatics.org/fasimu

for the latest updates and other information. There is also a project page at

http://www.bioinformatics.org/project/?group_id=1004

leading to bug tracking and a public forum.

8.2 License

FASIMU is published under the GNU public license (GPL) which can be viewed in a FASIMU session with

```
fasimu-license
```

There is also a license statement at the beginning of each of the source files. This document is likewise under the GPL.

Bibliography

[1] Bash, the GNU Project's Bourne Again SHell, a complete implementation of the IEEE POSIX and Open Group shell specification.

[2] J. S. Edwards, R. U. Ibarra, and B. O. Palsson. In silico predictions of escherichia coli metabolic capabilities are consistent with experimental data. *Nat Biotechnol*, 19(2):125–130, Feb 2001.

[3] Albert Gevorgyan, Mark G Poolman, and David A Fell. Detection of stoichiometric inconsistencies in biomolecular models. *Bioinformatics*, 24(19):2245–2251, Oct 2008.

[4] Sabrina Hoffmann, Andreas Hoppe, and Hermann-Georg Holzhütter. Pruning genome-scale metabolic models to consistent ad functionem networks. *Genome Inform*, 18:308–319, 2007.

[5] Hermann-Georg Holzhütter. The principle of flux minimization and its application to estimate stationary fluxes in metabolic networks. *Eur J Biochem*, 271(14):2905–2922, Jul 2004.

[6] Hermann-Georg Holzhütter. The generalized flux-minimization method and its application to metabolic networks affected by enzyme deficiencies. *Biosystems*, 83(2-3):98–107, 2006.

[7] Scott Holzhütter and Hermann-Georg Holzhütter. Computational design of reduced metabolic networks. *Chembiochem*, 5(10):1401–1422, Oct 2004.

[8] Andreas Hoppe, Sabrina Hoffmann, and Hermann-Georg Holzhütter. Including metabolite concentrations into flux balance analysis: thermodynamic realizability as a constraint on flux distributions in metabolic networks. *BMC Syst Biol*, 1:23, 2007.

[9] Carola Huthmacher, Andreas Hoppe, Sascha Bulik, and Hermann-Georg Holzhütter. Antimalarial drug targets in plasmodium falciparum predicted by stage-specific metabolic network analysis. *BMC Syst Biol*, 4(120), August 2010. accepted.

[10] Matthew D Jankowski, Christopher S Henry, Linda J Broadbelt, and Vassily Hatzimanikatis. Group contribution method for thermodynamic analysis of complex metabolic networks. *Biophys J*, 95(3):1487–1499, Aug 2008.

[11] Sarah Killcoyne, Gregory W Carter, Jennifer Smith, and John Boyle. Cytoscape: a community-based framework for network modeling. *Methods Mol Biol*, 563:219–239, 2009.

[12] Steffen Klamt, Julio Saez-Rodriguez, and Ernst D Gilles. Structural and functional analysis of cellular networks with CellNetAnalyzer. *BMC Syst Biol*, 1:2, 2007.

[13] J Küntzer, T Blum, A Gerasch, C Backes, A Hildebrandt, M Kaufmann, O Kohlbacher, and HP Lenhof. BN++ - a biological information system. *J Integr Bioinformatics*, 3(2):34, 2006.

[14] Francisco Llaneras and Jesús Picó. A procedure for the estimation over time of metabolic fluxes in scenarios where measurements are uncertain and/or insufficient. *BMC Bioinformatics*, 8:421, 2007.

[15] Jennifer L Reed and Bernhard Ø Palsson. Genome-scale in silico models of e. coli have multiple equivalent phenotypic states: assessment of correlated reaction subsets that comprise network states. *Genome Res*, 14(9):1797–1805, Sep 2004.

[16] Daniel Segrè, Dennis Vitkup, and George M Church. Analysis of optimality in natural and perturbed metabolic networks. *Proc Natl Acad Sci U S A*, 99(23):15112–15117, Nov 2002.

[17] Tomer Shlomi, Omer Berkman, and Eytan Ruppin. Regulatory on/off minimization of metabolic flux changes after genetic perturbations. *Proc Natl Acad Sci U S A*, 102(21):7695–7700, May 2005.

[18] Tomer Shlomi, Moran N Cabili, Markus J Herrgård, Bernhard Ø Palsson, and Eytan Ruppin. Network-based prediction of human tissue-specific metabolism. *Nat Biotechnol*, 26(9):1003–1010, Sep 2008.
