# Supplementary material for: FASIMU: flexible software for flux-balance computation series in large metabolic networks
Source: BMC Bioinformatics. 2011 Jan 22;12:28. doi: 10.1186/1471-2105-12-28 (PMC3038154; doi:10.1186/1471-2105-12-28)
Supplement: Additional file 1 — FASIMU 2.2.1 release archive. This archive contains the complete FASIMU distributions and unzips in five directories: FASIMU contains the programs, FASIMU-Doc contains the documentation (manual and tutorial), FASIMU-Ery-Example contains a small example of the human erythrocyte, FASIM-Ecoli-Example a large example of the E. coli, FASIMU-Liver-Example another large example of the human hepatocyte. [file 1471-2105-12-28-S1.ZIP › FASIMU_Doc/faBiNA_Manual.pdf]

faBiNA: Flux analysis plugin of BiNA

User manual

Introduction

The flux analysis plugin allows the input of a preformatted metabolic network file and enhances the function of BiNA. The functions of BiNA itself will not be documented here, please refer to

http://bina.sourceforge.net

and the help file within the graphical user interface if BiNA for documentation. There are other ways to input metabolic networks into BiNA (Küntzer, et al., 2006), however, the use of the plugin offers the following advantages:

- it can be controlled with a command line call of BiNA in a FASIMU session,
- the alias nodes (cofactor metabolites) can be defined with the option -a of allout2bina of FASIMU
- the colors of the compartment nodes can be defined with the -c option of allout2bina
- the input file can modified with a script inside a FASIMU session

The flux analysis plugin extends the BiNA functionality in the following ways:

- Different flux distributions in the same network can be viewed with a single mouse click
- Reactions with zero flux rate can be switched to visible or invisible
- The function by which the flux rate and the displayed thickness of reaction arrows can be modified
- The chemical structure of metabolites can be displayed next to their graph nodes.

Installation

BiNA is a software for visualizing regulatory and metabolic networks written in Java. Running BiNA requires at least Sun's JRE 1.6. You can download the current version from

http://www.java.com/en/download/

A pre-installed version of our FASIMU plugin in BiNA can be found at our sourceforge.net website at:

http://sourceforge.net/projects/binafluxmodel

Download the current version and extract the zip file (a separate folder will be created). If there was already any version of BiNA installed, delete the settings folder by removing the $HOME/.bina directory to avoid conflicts.

Test your installation by running one of the given start-up scripts: bina.sh for Linux users and BiNA.bat for Windows users. If it fails you should check your Java PATH entry or adjust the scripts at your needs.

ImporterWizard

In the left panel there is a wizard with the name “Flux balance analysis” which knows the steps to the import of a model file. The respective next step is activated by clicking the green forward arrow at the bottom. The first step chooses the file. The next steps presents three checkboxes:

- **Draw Pathwayboxes.** The graph is divided in subgraphs depending on the information in column 4 (see below) and each subgraph is drawn in a different box in the BiNA chart.
- **Draw Compartmentboxes.** The graph is divided in subgraphs depending on the information in column 4 (see below) and each subgraph is drawn in a different box in the BiNA chart.
- **Color Compartments.** The color respective to the compartment is not only used for the metabolite node boxes but also for the large surrounding boxes.

Functions of the FluxBalanceMapper

This is panel on the left hand side of the BiNA workspace. A click on it opens a properties pane which contains:

- the checkbox Hide Zero Fluxes. If checked the graph is drawn with out the reaction carrying no flux. Otherwise the zero fluxes are drawn.
- the checkbox Treat unknown fluxes as zero.
- a panel for the flux distributions. If several flux distributions have been given (see file format description below) they are displayed here and can be selected.
- Fields for the parameters controlling how the thickness of arrows depends on the flux rates.
    - **line max.** maximal line thickness.
    - **line min.** minimal line thickness.
    - **line high.** The line thickness corresponding to the flux rate in “high val”.
    - **line low.** The line thickness corresponding to the flux rate in “low val”.
    - **low val.** A flux value representing the lower bound of the range with the highest displayed resolution
    - **high val.** A flux value representing the upper bound of the range with the highest displayed resolution

Input file format

For the basic function of the plugin FASIMU creates this file for you and you will have to deal with the format only if you want to modify some of its elements.

The file has the extension csv and is organized in five sections A–E:

A. The first section contains the model.

The first line may hold a table head, it is simply ignored. Throughout this section lines starting with # are ignored as comments. The section can only be left by lines starting with

"Entering" or "Leaving". The lines inside this section are interpreted as reactions of the network, as follows. They are tab-separated:

Column 1: Reaction identifier

Column 2: Enzyme identifier

Column 3: Reaction equation

it is a format in which some of the models by Palsson group are published. It is obligatorily compartmentalized. Whitespace separated, with the exception of the compartment items (enclosed in brackets) which are directly tied to the next (Type 1 below) or previous (Type 2) item. The reaction arrow is one of <==> --> <-- (representing the irrversibility). The stoichimetric factors are enclosed in paranthesis and can be any float number. Factor 1 may be omitted. The metabolites identifiers are strings but the characters ()[]* although not forbidden may be misinterpreted and are discouraged. Each metabolite followed by an asterics marks it as a cofactor (called alias inside BiNA) of this reaction. The asterics is not part of the identifier (atp and atp* will refer to the same metabolite). In Type 2 (below) the asterics follows the compartment token.

There are two types in the notation of a reaction, one for one-compartment reactions, the other for transporters and multi-compartment processes

Type 1: The line starts with the compartment in brackets but without whitespace between it and the next item which can be an metabolite identifier or a stoichiometric coefficient. The metabolites are denoted without the trailing compartments. Example:

```
[c]f6p + atp* --> fdp
```

Type 2: All metabolites have a trailing compartment identifier enclosed in brackets. Example:

```
g3p[c] + atp[c]* --> g3p[e] + adp[c]* + pi[c]*
```

Column 4: Name of the pathway/reaction subsystem.

It is only interpreted if the drawPathway is ticked and drawCompartment is not ticked. If drawCompartment is ticked the compartment information is used for this.

Column 5: Gene association

Not used by BiNA currently

Note for the pathway/compartment annotations: reactions can be ordered in a two layer-model where the intermediate level may either be the compartment (drawCompartment ticked) or the pathway given in Column 4. Otherwise it is treated likewise. Reactions belonging to the same class (defined by the middle layer) are visualized in graphical blocks. Some reactions are set as belonging to no block, if the compartment/pathway token is "Transport", "Membrane", the empty string, or contains a "Y".

B. Entering metabolites section

This section is identified by the "Entering" keyword at the start of the line. Lines starting with # are ignored as comments. The respective metabolites recieve the setDescriptionAttribute("entering_metabolite") which gives the respective metabolite boxes a

special color. The metabolites must have trailing compartment in parenthesis (not brackets, as above). The section is ends when a line starts with "Leaving" or "Fluxmode".

C. Leaving metabolites section

This section is identified by a line beginning with "Leaving". It very similar to section B Lines starting with # are ignored as commments The respective metabolites recieve the setDescriptionAttribute("entering_metabolite") which gives the respective metabolite boxes a special color. The metabolites must have trailing compartment in parenthesis (not brackets, as above) The section is ends when a line starts with "Entering" or "Fluxmode".

D. Fluxmode(s) section

One or more flux modes are given in this section. This section is identified by a line beginning with "Fluxmode". If the identifier "Fluxmode" is found more than once, several flux modes are generated. The keyword fluxmode can be followed by a ":" and a name in which case this name is referred to in the FluxBalanceMapper. If not given a generic numbered name is used.

Each line containing a = is interpreted as a flux value, the LHS must refer to a reaction identifier the RHS must define a float number. The first line not containing a "=" or starting with # ends the flux mode. If the next line starts with "Fluxmode", the next fluxmode is read in, otherwise the section is cancelled. No blank lines or #-starting comments are allowed inside this section.

E. Parameters section

This section is identified by a line beginning with "Parameters" or "Defaults". It is scanned for lines containing a =. The LHS must be float number. The RHS is either the identifier or an item of an array (the parameter in parenthesis). Allowed identifiers are:

```
lowval highval linehigh linelow linemax linemin
```

Allowed functions are:

```
location_color(<compartment identifier>)
```

it takes a three dimensional vector of the form [R,G,B] where each entry is an interger from 0 to 255. The section is ended by the end of the file.

Example:

```
#ID     Mapping/Reaction name   reaction        subsystem
Eigene Gene Associations
1       HEX1    [c]glc-D + atp* --> g6p Glycolysis/Gluconeogenesis
glk
2       PGI     [c]g6p <==> f6p Glycolysis/Gluconeogenesis      pgi
3       PFK     [c]f6p + atp* --> fdp   Glycolysis/Gluconeogenesis
( pfkA  or  pfkB )
...
112     BM_g3p  g3p[c] --> BM_g3p[e]    Biomass Synthesis
artifical
#
Entering_metabolites:
glc-D(e)
o2(e)
```

```
no3(e)
#
Leaving_metabolites:
no2(e)
etoh(e)
#rID=flux
Fluxmode:
1=0
2=0.54
3=0
Parameters:
lowval=0.02
highval=1.0
linehigh=4.5
linelow=0.7
linemax=6.0
linemin=0.5
location_color(c)=[255,242,229]
location_color(m)=[204,153,255]
location_color(s)=[192,192,192]
```

References

Küntzer, J., Blum, T., Gerasch, A., Backes, C., Hildebrandt, A., Kaufmann, M., Kohlbacher, O. and Lenhof, H.P. (2006) BN++ – A Biological Information System, *J Integr Bioinformatics*, 3, 34.
