# Supplementary material for: FASIMU: flexible software for flux-balance computation series in large metabolic networks
Source: BMC Bioinformatics. 2011 Jan 22;12:28. doi: 10.1186/1471-2105-12-28 (PMC3038154; doi:10.1186/1471-2105-12-28)
Supplement: Additional file 1 — FASIMU 2.2.1 release archive. This archive contains the complete FASIMU distributions and unzips in five directories: FASIMU contains the programs, FASIMU-Doc contains the documentation (manual and tutorial), FASIMU-Ery-Example contains a small example of the human erythrocyte, FASIM-Ecoli-Example a large example of the E. coli, FASIMU-Liver-Example another large example of the human hepatocyte. [file 1471-2105-12-28-S1.ZIP › FASIMU_Doc/FASIMU_Tutorial.pdf]

Tutorial FASIMU

Andreas Hoppe - hoppe@bioinfomatics.org

November 25, 2010

This tutorial documents version 2.2.1.

Throughout the tutorial we assume that FASIMU is properly installed and CPLEX is available as the solver. If another solver is installed, CPLEX specific parts might differ. It is also assumed that the UNIX tools `wget`, `unzip` (GNU zip), `diff`, `wc grep`, and less and BiNA are installed along with the visualization program BiNA and the plugin faBiNA.

Chapter 1

Plethora of FASIMU functions demonstrated on a small human erythrocyte model

Introduction

In this tutorial we perform flux-balance computation on a model of the energy and redox metabolism of human red blood cells [6] thereby learning the internal structure of a FASIMU session and applying several of the implemented algorithms.

Preparation

Install FASIMU together with a solver and BiNA as described in the manual. Download the file `FASIMU_Ery_Example.zip` for example with the command-line tool `wget`:

```
cd ~
mkdir FASIMU-Test
cd FASIMU-Test
wget http://www.bioinformatics.org/fasimu/FASIMU_Ery_Example.zip
unzip FASIMU_Ery_Example.zip
sbml2fa ery.sbml
```

Of course, `wget` can be exchanged with `links`, `lynx` or some other http-capable prgram or you can download the file with an internet browser.

Getting acquainted

As you may have learned in the manual all FASIMU files are human-readable text files. Several files have been generated from the SBML file which will be used for the simulations. The SBML file will not be regarded any more. So to start with it is a good idea to have a look at the information in the files:

```
less reactions
less metabolites
less equilibriums
```

```
less enzymes
less simulations
```

Getting started

Start FASIMU with

```
source fasimu
```

A status message appears at the screen including the number of reactions and metabolites. Note that some files have changed now:

```
less reactions
less metabolites
```

Pseudo metabolites ending with _ex and pseudo reactions whose identifier ends with _tr have been added. This is necessary to have full control: the simulations environment can allow the excretion and uptake of any metabolite in the model even if it is not defined to be an exchange metabolite in the original model. Usually you can ignore this but it is important to have this in mind if you analyze intermediate information files. The repeated call of source fasimu does not add these pseudo reactions again: the files ending with .original, for instance

```
less reactions.original
```

keep the original files. So it is safe to call source fasimu in the same directory again.

The solver has been chosen automatically. If an error message about the solver is displayed the installation of the solver on your system might not be correct. The solver can be manually selected with source fasimu <solver>, where <solver> can be cplex10, cplex9, glpk, lp_solver, or lindo.

Before any simulation is started the FBA function call together with options can be chosen by setting the variable optimization_call. Otherwise the default is chosen:

```
Info: variable 'optimization_call' set to the default of "compute-FBA".
```

referring to the flux minimization [4].

Perform the simulations

The simulation tasks are predefined in the file simulations:

```
cat simulations
```

You will see the definition of the simulations: e.g. in the second column (the objective) and the third column (the constraints). The identifier stdef among the constraints refers to additional constraints in a separate file (the file extension .txt may be omitted).

```
cat stdef.txt
```

This is a convenient way to define substrates and waste products. The result of the substitution can be checked in a another file:

```
cat simulations_work.fgf
```

You may notice that here all the substrates and waste products are mentioned again which have already been marked as exchange metabolites in the original SBML file. In SBML, inward and outward fluxes are not differentiated by the `boundaryCondition` variable in the `reaction` tag. Therefore, they are ignored in FASIMU. However, it is possible to use the original boundaryCondition information in a file with the function call

```
make-standard-exchangables > autostdef.txt
cat autostdef.txt
```

In contrast to the above file `stdef.txt`, here the exchange is allowed in both directions (marked with a = character).

Start the simulations with:

```
simulate
```

Note the progress information, each simulation computed is acknowledged by giving the number of simulations computed already and the total number of simulations. This is an especially useful feature for larger models, computationally harder algorithms, and a long series of simulations. The rest of the line gives an overview of the computed result which is also written to the file `evaluation.txt`.

View the results in text form

The overview of all computed results can be displayed with

```
cat evaluation.txt
```

You may notice the + characters in the second column indicating that the simulation is successful, e.g. in some cases the production of a certain metabolite, in other cases the non-existence of a solution. The fourth column in the file `simulations` is devoted to this question: a metabolite identifier means that this metabolite must be exchanged over the system boundary. It is also possible to give a reaction identifier there (in which case this reaction must carry a nonzero flux) or a zero indicating that the simulation is considered to be successful if no solution exists.

```
cut -f1,4 simulations
```

For long lists of simulations simplified function is supplied which only shows the failed simulations:

```
negeval
```

In the given example nothing is shown; all simulations show the desired result. The actual flux distributions predicted are contained in `allout.txt`:

```
less allout.txt
```

This file shows all flux distributions of a computation series. The flux distributions are separated by lines with many `#` characters followed by the name of the simulation. In the next line there is a description of the system boundaries of this particular simulation. The next line gives a short comment whether a solution is computed with optional comments from the solver. Additionally warnings are shown here.

Then the solution is given in a tab-separated format. The first column gives the name of the reaction. The second column gives the flux value. Note that zero fluxes are not recorded in the solution. Next column gives the equilibrium constant just for information, then the reaction written with metabolite identifiers follows. FASIMU can also be ordered to write them with metabolite names given in the file metabolites: most conviniently this can be done in the initial sartup of FASIMU:

```
source fasimu names_in_allout
```

The fourth column hold possible reaction names given in the file `reaction-names`.

For computations where the thermodynamic feasibility [5] is checked a section follows given hypothetical concentrations which are compatible with the flux distribution.

View the results in the visualization packages

Using BiNA

The flux analysis plugin of BiNA requires a special input file which can be generated from `allout.txt` with the function `allout2bina`. The call `bina` is defined in FASIMU and is not the normal way to start BiNA. The main advantage using the function `bina` in FASIMU is that the flux analysis plugin is automatically invoked and the flux mode file is loaded.

```
allout2bina
bina BiNA/All.csv
```

See BiNA manual (http://bina.sourceforge.net/documentation) how to work with BiNA and the faBiNA manual (http://www.bioinformatics.org/fasimu/faBiNA_Manual.pdf) how to work with the flux analysis plugin!

You may notice two things. The node ATP is highly connected and so are the nodes of ADP, NAD, and NADH. You can set the alias function for such metabolites one by one in BiNA. However fasimu offers an easy way to define some metabolites as aliases right from the start:

```
allout2bina -a ATP ADP NAD NADH
```

Loading the result file in bina as above the nodes of these metabolites are hidden. The BiNA function View→Open children makes them visible but with instances.

You may also notice that the exchange metabolites are colored (reddish for input and orange for output) and the internal metabolites have no color. But it is possible to assign colors to metabolites depending on the compartment:

```
allout2bina -c cell[49,234,222] ext[244,44,238]
```

The syntax is straightforward: first the compartment name and then the RGB code in brackets with values ranging from 0 to 255. No space characters are allowed inside the compartment/RGB item.

Using Cytoscape + FluxViz

First, prepare the flux distribution files:

```
allout2valfiles
```

Call Cytoscape, read the file ery.sbml with the plugin SBMLReader2 and read in the val-files. See the manual of FluxViz (http://www.charite.de/sysbio/people/koenig/software/fluxviz/help/index.html#Usage) for more details.

Using CellNetAnalyzer

The flux distribution files are again prepared with:

```
allout2valfiles
```

Copy the files reactions and metabolites into a separate order and create a CellNetAnalyzer model (see CellNetAnalyzer manual for details). Start MatLab and CellNetAnalyzer in the MatLab command window, then load the model just created. Load the val-files with the Option called "CellNetAnalyzer→Read scenario".

Further algorithms

Thermodynamic feasibility

This constraint [5] requires equilibrium constants in the file `equilibriums` and concentration ranges. The latter are defined for metabolite classes for the benefit of easy management. The classes are defined with `concentration-ranges-classes` and each class has common concentration ranges given in `concentration-class-ranges`. Each line holds the strict lower bound, the relaxable lower bound, the envsioned set point, the relaxable upper bound, and the strict upper bound. First, you might to review this information

```
cat equilibriums
cat concentration-class-ranges
cat concentration-ranges-classes
```

Then, start the computation series

```
optimization_call="compute-FBA -T"
simulate
less allout.txt
```

The option `-T` selects the TR-criterion to be used with default parameters. In `allout.txt` you will see not only the reaction but also concentration values compatible with this flux distribution. For more realistic concentration values you may want to switch on the soft bounds (`-s` option) and the set points (`-w`) option, the latter only available for CPLEX (quadratic objective).

```
optimization_call="compute-FBA -T -w 1 -s 100"
simulate
less allout.txt
```

By default, only the hard bounds are used.

Now we might be interested in the concentration prediction, say for intracellular NAD. We see the concentration values of all computed solutions in the file `allout.txt`:

```
grep ^NAD_cell allout.txt
```

We see that the predicted concentration differs from the given set point. This time we want to put more emphasis on the set point and we can do this by attaching a weight to the concentration value (see manual for all the possibilities and details):

```
mv concentration-class-ranges c
gawk '$1=="NAD"{$4="0.0654e-3(20)"}{print}' c > concentration-class-ranges
update-concentration-ranges
optimization_call="compute-FBA -T -w 1 -s 100"
simulate
grep ^NAD_cell allout.txt
```

To restore it to the old concentration file the folling command do that:

```
cp c concentration-class-ranges
update-concentration-ranges
```

Intake minimization

As opposed to the previous simulations here only the usage of glucose is minimized, thus, calculating the maximal yield with respect to substrate utilization:

```
echo -e "ATPase 2.38\nDPGase 0.494\n\
GSHox 0.093\nPPrPT 0.0258" > setfluxes
optimization_call="compute-FBA"
echo -e "GlcT-min\tmin GlcT\tstdef\tGlcT" >> simulations
simulate-single GlcT-min
rm setfluxes
```

You can visualize the results of the simulation as above.

Pruning

To remove reactions which can not carry a non-zero flux and to fix the directions of fluxes which can not proceed in the other direction [3] you do the following. The system boundaries must be written in the file with the fixed name `stdexch.txt`.

```
cp stdef.txt stdexch.txt
optimization_call="compute-FBA -F 0"
prune-network
```

Here the most simple flux minimization protocol is used to test whether a reaction can carry a nonzero flux in one of the directions. However it is also possible to combine pruning with any other computational protocol. The result is stored in the directory sub:

```
(cd sub, cat reactions)
```

You can review the flux distributions computed to infer the pruned network — they are stored in `allout.txt` as usual. Note that the first flux distribution already yielded the result for most of the reactions. Most computation haven been done to verify that the backward direction of certain fluxes is not feasible.

The prune function writes its own `simulations` file, thus the previous contents is lost. To continue withthe tutorial, its previous contents is reloaded from the archive:

```
unzip FASIMU_Ery_Example.zip simulations
```

Expression-based activity prediction

This algorithm maximizes the number of common occurrence of flux and expression. First, we generate a random profile:

```
gawk '{print $1,rand()}' reactions.original > expressions
```

Constraints and objectives are not used by this algorithm thus, we add a "dummy" simulation and then run with:

```
optimization_call="compute-shlomi"
echo -e "Shlomi\tShlomi\t\tGlcT" >> simulations
simulate-single Shlomi
```

In FASIMU it also possible to combine the expression based-matching with any other flux optimization:

```
optimization_call="compute-FBA -x"
simulate-single All
```

See the manual for more parameters which control this feature.

Note that `simulate-single` appends to `allout.txt` and `evaluation.txt` whereas `simulate` deletes them before it starts to record results in.

Biomass maximization

As opposed to flux minimization where the input of substrates may not be bounded, in biomass maximization this is necessary for the problem to be feasible. We define this with this mechanism:

```
echo GlcT 0 1 > fluxbounds
update-fluxbounds
```

Again, we have to define a dummy simulation task because there is no fixed objective here.

```
optimization_call="compute-FBA"
echo -e "Biomass-max\tmax ATPase GSHox\tstdef\tGlcT" >> simulations
simulate-single Biomass-max
```

To reset the model we remove the fluxbounds and call a update function

```
rm fluxbounds
update-fluxbounds
```

See manual for a complete description of the update functions.

FVA

As for biomass maximization, for flux-variability analysis it is important to restrict the exchange fluxes otherwise each flux can grow infinitely. Here, the output is fixed:

```
echo -e "ATPase 0 2.38\nDPGase 0 0.494\nGSHox 0 0.093\nPPrPT 0 0.0258" > fluxbounds
make-FVA-simulationsfile > simulations
cp stdef.txt stdexch.txt
optimization_call="compute-FBA"
simulate
allout2valfiles
FVA-valfiles-chart
rm -rf val fluxbounds
update-fluxbounds
```

MFA

In metabolic flux analysis some fluxes have been experimentally measured and the rest is predict via the flux-balance condition. In FASIMU it can be realized with the `setfluxes` feature. We perform a FVA as above to see whether the system has still degrees of freedom which should be reduced with further measurement. Say, in the first measurement the import of Glucose and the excretion of lactate and carbon dioxid has been measured:

```
echo -e "GlcT 1.13\nCO2T 0.12\nLacT 1.34" > setfluxes
make-FVA-simulationsfile > simulations
cp stdef.txt stdexch.txt
simulate; allout2valfiles; FVA-valfiles-chart; rm -rf val
```

We see that many fluxes are still not determined. If additionally the export of pyruvate is measured:

```
echo "PyrT 0.34" >> setfluxes
simulate; allout2valfiles; FVA-valfiles-chart; rm -rf val
```

There is still a degree of freedom regarding the three fluxes DPGase, ATPase, and DPGM. Measuring DPGM yields:

```
echo "DPGase 0.04" >> setfluxes
simulate; allout2valfiles; FVA-valfiles-chart; rm -rf val
```

Now the system is completely determined. Note that this procedure requires a quite few flux optimizations and an analysis of the flux space with elementary flux modes or extremal pathways seems to be superior. However, for large systems a linear program is still feasible and in fact fast computable whereas the topological problems become practically infeasible.

Perturbation prediction by MOMA or ROOM

These predictions are based on a reference solution in an unperturbed state which is computed by:

```
unzip FASIMU_Ery_Example.zip simulations
simulate-single All
cp solution.txt refsolution.txt
```

Next we generate new simulations by the single knock out of a different reaction:

```
gawk -v OFS="\t" '{print $1"-inhib","","stdef","1"}' reactions.original > simulations
```

The function call for MOMA is compute-moma. See the manual for options and a detailed description.

```
optimization_call="compute-moma"
simulate
```

The function call for ROOM, the binary version of MOMA, is compute-room. See the manual for options and a detailed description.

```
optimization_call="compute-room refsolution.txt"
simulate
```

For CPLEX Version≥10 the implementation with conditionals is recommended:

```
optimization_call="compute-room refsolution.txt -c"
simulate
```

Here, we use the parameters given by Shlomi for lethality predictions.

```
optimization_call="compute-room refsolution.txt -c -d 0.1 -e 0.01"
simulate
```

Chapter 2

Tutorial: From a published Genome-scale network to computations

Introduction

This tutorial is dedicated to the process to make an abritrary network, published as SBML and a few additional information tables ready for computation in FASIMU. In the previous tutorial everything was prepared ready for FASIMU, but here we start directly from published files. So the first part of this tutorial chapter as rather a tutorial on the work with `bash` and `gawk` to transform networks than on FASIMU. However, since FASIMU is based on bash and gawk it might put you in the position to better understand the source code of FASIMU.

Feist et al. [1] published a highly recognized large network of *E. coli.* We start from two SBML files `Ec_iAF1260_flux1.xml` and `Ec_iAF1260_flux2.xml` each giving the same network but a different flux solution and a spreadsheet text files saved from the excel document `msb4100155-s3-reactions.tsv`.

For convinience I put the files together with some scripts in the file `FASIMU_Ecoli_Example.zip` downloadable from the FASIMU website. To start call

```
mkdir -p ~/ecoli-test
cd ~/ecoli-test
rm -f *
wget http://www.bioinformatics.org/fasimu/FASIMU_Ecoli_Example.zip
unzip FASIMU_Ecoli_Example.zip
```

To extract to network and the flux solution from the SBML we use the FASIMU script sbml2fa and a gawk line:

Extract the information from sbml and suppl files

```
sbml2fa Ec_iAF1260_flux1.xml
 for i in 1 2; do
gawk 'match($0,/<reaction id="([^"]+)"/,A){r=A[1];next}
match($0,/<parameter id="FLUX_VALUE" value="([^"]+)"/,A){if (A[1]!=0)print r,A[1];next}
' Ec_iAF1260_flux$i.xml > flux$i.val
done
```

The supplementary text of the reaction have been downloaded just for the Gibb's free energy values which are used to compute the equilibrium constants, using room temperature. First you can review the little script which does that, then you execute it.

```
cat calculate_equilibriums
source calculate_equilibriums
```

Now we got all the information together but there are some issues which would make the further work of FASIMU unnecessarily inconvinient. To make this process more transparent we rename the affected files and give them an extension to indicate the action number.

```
for f in reactions metabolites TR-excluded equilibriums flux1.val flux2.val; do
  mv $f $f.1
done
```

Action 1: Removing trailing _R _M

The first issue is that each metabolite identifier begins with a `M_` and each reaction identifier with a `R_`. This might be useful for other applications here it makes the identifier longer than necessary. To increase the readablity of the text files we remove them:

```
gawk '{
  if (!match($1,/^R_(.+)$/,A)) {print "Error">"/dev/stderr";exit}
  $1=A[1];
  i=2;
  while($i!="|") {
    if (match($i,/^M_(.+)$/,A)) $i=A[1];
    i++;
  }
  print
}' reactions.1 > reactions.2

gawk '{
  for (i=1;i<=2;i++) if (match($i,/^M_(.+)$/,A)) $i=A[1];
  print
}' metabolites.1 > metabolites.2

for f in  TR-excluded equilibriums flux1.val flux2.val; do
  gawk '{if (!match($1,/R_(.+)/,A)) {print "Error">"/dev/stderr";exit}
    $1=A[1];
    print
  }' $f.1 > $f.2
done
```

Action 2: Rename compartments

Next we recognize bulky compartment identifiers attached to the metabolites suc has `_c_Cytosol`. They come from the fact that the original identifiers as SBML-IDs have the `_c` attached, the compartment as the SBML-tag was called `Cytosol`. The script sbml2fa did not recognize it to be identical and therfore added `_Cytosol` to each cytosolic identifier. We remove them in all files containing metabolite identifiers.

```
for f in reactions metabolites; do
  gawk '{gsub(/_p_Periplasm/,"_p");
```

```
    gsub(/_e_Extraorganism/,"_e");
    gsub(/_c_Cytosol/,"_c");
    print
}' $f.2 > $f.3
done
```

The rest of the files are simply copied.

```
for f in TR-excluded equilibriums flux1.val flux2.val; do cp $f.2 $f.3; done
```

Action 3: Remove systems boundary

```
gawk 'match($1,/^(EX|DM)_/){print "="$3}' reactions.3 > stdexch.txt.4
for i in 1 2; do
  gawk 'BEGIN{D="reactions.3";while((getline<D)>0)T[$1]=$3;close(D)}
match($1,/^(EX|DM)_/){print ($2<0?"-":"+") T[$1]}' flux$i.val.3 > flux"$i"exch.txt.4
done

gawk '{
  if (match($1,/_b_(Extraorganism|Cytosol)$/)) next;
  print
}' metabolites.3 > metabolites.4

for f in reactions TR-excluded equilibriums flux1.val flux2.val; do
  gawk '{if (match($1,/^(EX|DM)_/)) next;
    print
  }' $f.3 > $f.4
done
```

Action 4: Restore the names

The transformation work is finished, the original names are restored:

```
for f in reactions metabolites TR-excluded equilibriums flux1.val flux2.val \
   stdexch.txt flux1exch.txt flux2exch.txt; do
  mv $f.4 $f
done
```

Action 5: Correct the equilibrium constants

The obedience of the equilibrium constants to the WEGSCHEIDER condition is checked (is not stricly obeyed) and be forced (with minimal modification):

```
source fabase
well-formed-equilibriums
cp corrected-equilibriums equilibriums
```

Wide default concentration ranges for TR

For a basic use of TR, wide concentration ranges suffice:

```
gawk '{print $1,"default"}' metabolites > concentration-ranges-classes
echo "default 10e-9 1e-6 50e-6 1e-3 100e-3" > concentration-class-ranges
```

Simlations file for biomass production

Based on comments in the data the availiabilty of glucose is set as follows to define the simulations:

```
echo -e "Biomass1\tEc_biomass_iAF1260_core_59p81M\tstdexch\t1\t\n\
Biomass-max1\tmax Ec_biomass_iAF1260_core_59p81M\tflux1exch -8 <= glc_D <= 0\t1\t\n\
Biomass-max2\tmax Ec_biomass_iAF1260_core_59p81M\tflux2exch -11 <= glc_D <= 0\t1\t\
" > simulations-ref;
```

Computations

Check the biomass flux predicitons

```
cp simulations-ref simulations
source fasimu
default_compartment=e
optimization_call="compute-FBA"
simulate
```

To do the same calculation with thermodynamic realizability [5]:

```
optimization_call="compute-FBA -T"
rm cplex-times.txt
time simulate
gawk '{s+=$4}END{print s"s"}' cplex-times.txt
```

Here we compared the total FASIMU running time with the time internally measured in CPLEX to show that the overhead time of FASIMU small compared with the solver time.

Full producibility test

Check the producibility for all internal metabolites using flux minimization with and without thermodynmic realizability [5]:

```
make-fullproducibility-simulationsfile > simulations
optimization_call="compute-FBA"
rm cplex-times.txt
time simulate
gawk '{s+=$4}END{print s"s"}' cplex-times.txt
negeval | wc -l
```

```
optimization_call="compute-FBA -T"
rm cplex-times.txt
time simulate
gawk '{s+=$4}END{print s"s"}' cplex-times.txt
negeval | wc -l
```

Degradablity test

Likewise degradability:

```
make-fulldegradibility-simulationsfile > simulations
optimization_call="compute-FBA"
rm cplex-times.txt
time simulate
gawk '{s+=$4}END{print s"s"}' cplex-times.txt
negeval | wc -l

optimization_call="compute-FBA -T"
rm cplex-times.txt
time simulate
gawk '{s+=$4}END{print s"s"}' cplex-times.txt
negeval | wc -l
```

Leak analysis

Perform a leak analysis

```
make-leakcheck-simulationsfile > simulations
rm cplex-times.txt
optimization_call="compute-FBA -F 0"
time simulate
gawk '{s+=$4}END{print s"s"}' cplex-times.txt
negeval
```

Pruning

Pruning with the defintion of the exchange fluxes as given above. One metabolite was missing from the orignally defined systems boundary: the actual biomass which is added to `stdexch.txt`.

```
echo "+Ec_biomass_iAF1260_core_59p81M" >> stdexch.txt
rm cplex-times.txt
optimization_call="compute-FBA -F 0"
time prune-network
gawk '{s+=$4}END{print s"s"}' cplex-times.txt
wc -l cplex-times.txt
wc -l sub/reactions
diff reactions sub/ | grep ^< | wc -l
```

Chapter 3

Using FASIMU for network curation

Introduction

Here, a recently published network of the human hepatocyte [2] is used, given with the simulations to verify it:

```
wget http://www.bioinformatics.org/fasimu/FASIMU_Liver_Example.zip
unzip FASIMU_Liver_Example.zip
sbml2fa liver.sbml
default_compartment=ext
source fasimu names_in_allout
```

This network is considerably larger than the published network since it contains many reactions not feasible under steady-state conditions. Think of it as an intermediate stage in a network reconstruction process where the metabolic functions are described in `simulations` and the system boundaries (substrates, excretion products, and biomass components) are contained in the file `stdexch.txt`.

Pruning to the functional network

Blocked reactions are removed with respect to the defined systems boundary. Additionally, if a single direction of the reaction is blocked it is marked as irreversible in the appropriate direction.

```
prune-network
cd sub
unzip ../FASIMU_Liver_Example.zip simulations MIMES.txt MIPES.txt PIPES.txt
```

Performing the simulations with plain FBA

```
optimization_call="compute-FBA"
simulate
negeval
```

Some simulations fail which results from the fact that some evalutors check for conditions of thermodynmically infeasible states.

Performing the simulations with thermodynamic feasibility

```
optimization_call="compute-FBA -T -c"
simulate
negeval
```

Here also the post check on the returned flux solution is preformed (`-c` option). Where applicable, results apear as warnings in `evaluation.txt`:

```
cat evaluation.txt
```

Leak analysis

Perform a leak analysis

```
make-leakcheck-simulationsfile > simulations
rm cplex-times.txt
optimization_call="compute-FBA -F 0"
time simulate
gawk '{s+=$4}END{print s"s"}' cplex-times.txt
negeval
```

Bibliography

[1] Adam M Feist, Christopher S Henry, Jennifer L Reed, Markus Krummenacker, Andrew R Joyce, Peter D Karp, Linda J Broadbelt, Vassily Hatzimanikatis, and Bernhard Ø Palsson. A genome-scale metabolic reconstruction for escherichia coli k-12 mg1655 that accounts for 1260 orfs and thermodynamic information. *Mol Syst Biol*, 3:121, 2007.

[2] Christoph Gille, Christian Bölling, Andreas Hoppe, Sascha Bulik, Sabrina Hoffmann, Katrin Hübner, Anja Karlstädt, Ramanan Ganeshan, Matthias König, Kristian Rother, Michael Weidlich, Jörn Behre, and Herrmann-Georg Holzhütter. Hepatonet1: a comprehensive metabolic reconstruction of the human hepatocyte for the analysis of liver physiology. *Mol Syst Biol*, 6:411, Sep 2010.

[3] Sabrina Hoffmann, Andreas Hoppe, and Hermann-Georg Holzhütter. Pruning genome-scale metabolic models to consistent ad functionem networks. *Genome Inform*, 18:308–319, 2007.

[4] Hermann-Georg Holzhütter. The principle of flux minimization and its application to estimate stationary fluxes in metabolic networks. *Eur J Biochem*, 271(14):2905–2922, Jul 2004.

[5] Andreas Hoppe, Sabrina Hoffmann, and Hermann-Georg Holzhütter. Including metabolite concentrations into flux balance analysis: thermodynamic realizability as a constraint on flux distributions in metabolic networks. *BMC Syst Biol*, 1:23, 2007.

[6] R. Schuster and H. G. Holzhütter. Use of mathematical models for predicting the metabolic effect of large-scale enzyme activity alterations. application to enzyme deficiencies of red blood cells. *Eur J Biochem*, 229(2):403–418, Apr 1995.
