# Supplementary material for: FASIMU: flexible software for flux-balance computation series in large metabolic networks
Source: BMC Bioinformatics. 2011 Jan 22;12:28. doi: 10.1186/1471-2105-12-28 (PMC3038154; doi:10.1186/1471-2105-12-28)
Supplement: Additional file 1 — FASIMU 2.2.1 release archive. This archive contains the complete FASIMU distributions and unzips in five directories: FASIMU contains the programs, FASIMU-Doc contains the documentation (manual and tutorial), FASIMU-Ery-Example contains a small example of the human erythrocyte, FASIM-Ecoli-Example a large example of the E. coli, FASIMU-Liver-Example another large example of the human hepatocyte. [file 1471-2105-12-28-S1.ZIP › FASIMU_Doc/FASIMU_upgrade_guide.pdf]

FASIMU upgrade guide

This guide applies to FASIMU 2.1 respectively FABASE 3.0 .

In preview of the publication of FASIMU a considerable number of functions and files have been changed.

`fa2lpf` is now called `fabase`, the former was too hard to pronounce. This change involves the file fa2lpf and some of the functions: fa2lpf-functions fa2lpf-help.

New unified guidelines of the user interface

File contents

All files are whitespace-separated text files except where a two level separation is necessary (simulations) in which case the primary separater is the tab and the secondary sequences of space characters.

File extensions

Files with the original extension are backups of the original files without extension.

All files to be supplied and modified by the user are text files with no extension.

All files produced by FASIMU being of interest to the user have the extension txt.

All files produced and internally used by FASIMU being of lesser interest to the user have the extension fgf.

All files produced used a parts of the problem formulation have the extension lpf.

Output of external programs (and derivates therof) carries the extension out.

Input to external programs carries the extension in.

The extension lp refers to the CPLEX-LP format, the extension ltx to the so-called LINDO format.

The extension par refers to parameter files of LINDO and lp_solve.

Function names

Function names (now) carry dashes and not underline characters (except if it contains the identifier lp_solve which is dictated by the name of the solver)

File parameter

Functions do not accept file parameters, instead the files used have fixed names. If several files for the same purpose are used throughout a session, the session script must rename them.

The respective options are switched on by the existence of these files, e.g. enzymes are considered if the file `enzymes` exists.

File overwrite

Main user files (indicated by no extension) are mostly not overwritten. One notable exception is the `source fasimu` call which modifies reactions, metabolites, equilibriums, targetfluxes where the original files are restored in the respective files with the extension .original.

The parameter files (cplex-head.in and files with the extension par) are only created by source fabase / fasimu if not present. Present files will only be overwritten if explicitly requested by the restore-default-parameters function.

FABASE changes

Changes for the solver call

The solvers LINDO, lp_solve, GLPK are now alternatives to CPLEX: Thus the core computation functions have changed their names: now solver-std-call is the computation gateway, independent from the selected solver. The new function FB-optimization-std-call uses this function and is the gateway for Flux optimization functions.

solver-std-call dispatches to the solver-specific functions cplex-std-call, lindo-std-call, lp_solve-std-call, glpk-std-call. All above functions now have an exit value, zero indicates a successful run, 1 a infeasible, unbounded or malformed problem.

The former cplex.in is replaced by cplex-head.in, cplex-tail.in and problem.lp. The latter is the solver-independent optimization problem file in CPLEX-LP format which can be read directly in lp_solve and GLPK. For LINDO there is a function cplexlp2lindoltx which compiles this file to the LINDO-LP format.

The former cplex.out is replaced by solver.out which is also the solver output for the other solvers. It does not contain the solution anymore, instead, all the variables returned by the optimizer are compiled to a solver independent file variables.txt which is filtered to create a file for the flux values (solution.txt) and concentrations (concentrations.txt).

Changes for the flux optimization

There is a single portal function compute-FBA replacing the former functions, which are still available, but the dispatch to the new function.

Old function	new funciton
compute-fluxmin x	compute-FBA -F x
compute-fitness-fluxmin	compute-FBA -f ...
compute-minintake x	compute-FBA -m x
compute-fitness-minintake x	compute -m x -f ...
compute-TR-FBA x	compute-FBA -T x

The former fluxmin.out is replaced by solution.txt because FABASE also supports (biomass) maximization and substrate minimization. This file contains the reaction identifier to the value, as opposed to the out-format, which contained the internally numbered reaction identifier. The latter format will not be relevant to the user anymore, however, the solution.out in the same format as fluxmin.out is still created.

The type identifiers for the TR computation have changed. The former types A,C,D have been deleted because they are neither biochemically founded nor technically useful.

Former type	new type	short description

A	deleted	
B	a	zero potential – any flux, zero flux – any potential
C	deleted	
D	deleted	
E	b	zero potential – zero flux
F	deleted	
G	A	same as a but with the conditional feature of cplex
H	deleted	
I	deleted	
J	B	same as b but with the conditional feature of cplex

The default has not changed: it was B respectively G for solver CPLEX version>=10 before and is now a and A respectively.

FASIMU changes

The file simulations.txt is now called simulations (although the old name is still supported) to convey to the new file extension policy.

In all function names the underline character is replaced with a dash (see below). The old function names are available by calling the function `fasimu-old-functions-aliases`.

The function allout-select changes its specification when no second argument is given. Now it reads by default from the file allout.txt. The same is true for allout-summarize.

The variable `$fluxmin_call` has been renamed to `$optimization_call`.

The file allout.txt now prints out the reaction using the metabolite identifiers. Previously it used the names of the metabolites given in the file `metabolites`. This affects also the files given to the BiNA output.

The function `simulations` is now called `simulate` (to avoid problems and confusion with the file `simulations`). The function `simulations_single` is now called `simulate-single`

Many of the functions are very specific to certain projects, are not documented in the manual and may be deleted in the future.

Old function	New function	Comment
allout2bina_hepato2	allout2bina-hepato2	HepatoNet-specific
allout2bina_hepato3	allout2bina-hepato3	HepatoNet-specific
allout2sabina_matrix	allout2sabina-matrix	
allout2sabina_model	allout2sabina-model	
allout2sbml21_files	allout2sbml21-files	
allout2wholebina_hepato2	allout2wholebina-hepato2	HepatoNet-

		specific
allout_check_unbalanced	allout-check-unbalanced	
allout_filter_boundary_reactions	allout-filter-boundary-reactions	
allout_prefilter	allout-prefilter	
allout_select	allout-select	
allout_summarizer	allout-summarizer	
allout_transform_to_one_letter_compartments	allout-transform-to-one-letter-compartments	HepatoNet-specific
alloutitem2sabina_model_with_fluxval	alloutitem2sabina-model-with-fluxval	
alloutitem2sabina_model	alloutitem2sabina-model	
alloutitem2sabina_na	alloutitem2sabina-na	
alloutitem_check_unbalanced	alloutitem-check-unbalanced	
fa2sabina_model	fa2sabina-model	
fasimu_main	fasimu-main	
last_simulation	last-simulation	
make_FVA_simulationsfile	make-FVA-simulationsfile	
make_alloutdir	make-alloutdir	
make_delocmet	make-delocmet	
make_fullproducibility_simulationsfile	make-fullproducibility-simulationsfile	
make_futileproducibility_simulationsfile	make-futileproducibility-simulationsfile	
make_leakcheck_simulationsfile	make-leakcheck-simulationsfile	
make_rawmet	make-rawmet	
make_sbmls	make-sbmls	
make_standard_exchangables	make-standard-exchangables	
make_standard_wastables	make-standard-wastables	
make_targetexchanges_simulationsfile	make-targetexchanges-simulationsfile	
make_targetfluxes_simulationsfile	make-targetfluxes-simulationsfile	
make_valfiles	make-valfiles	
make_wastability_simulationsfile	make-wastability-simulationsfile	
prune_network_create_model	prune-network-create-model	

prune_network_simple	prune-network-simple	
prune_network	prune-network	
sabformat2sbml21_files_with_exmetabolites	sabformat2sbml21-files-with-exmetabolites	
sabformat2sbml21_files	sabformat2sbml21-files	
sabina_model_refine_hepato	sabina-model-refine-hepato	HepatoNet-specific
sabina_model_refine_hepato2	sabina-model-refine-hepato2	HepatoNet-specific
sabina_model_refine_plasmodium	sabina-model-refine-plasmodium	
sabina_model_refine_yeast	sabina-model-refine-yeast	
sabina_transform_to_one_letter_compartments	sabina-transform-to-one-letter-compartments	HepatoNet-specific
select_from_allout	allout-select	
simulations_noallout	simulate-noallout	
simulations_single_wrap	simulate-single-wrap	
simulations_single	simulate-single	
simulations_ana	simulations-ana	
simulations_work_assure	simulations-work-assure	
zeval	negeval	
