# Supplementary figures and images for: FASIMU: flexible software for flux-balance computation series in large metabolic networks
Source: BMC Bioinformatics. 2011 Jan 22;12:28. doi: 10.1186/1471-2105-12-28 (PMC3038154; doi:10.1186/1471-2105-12-28)

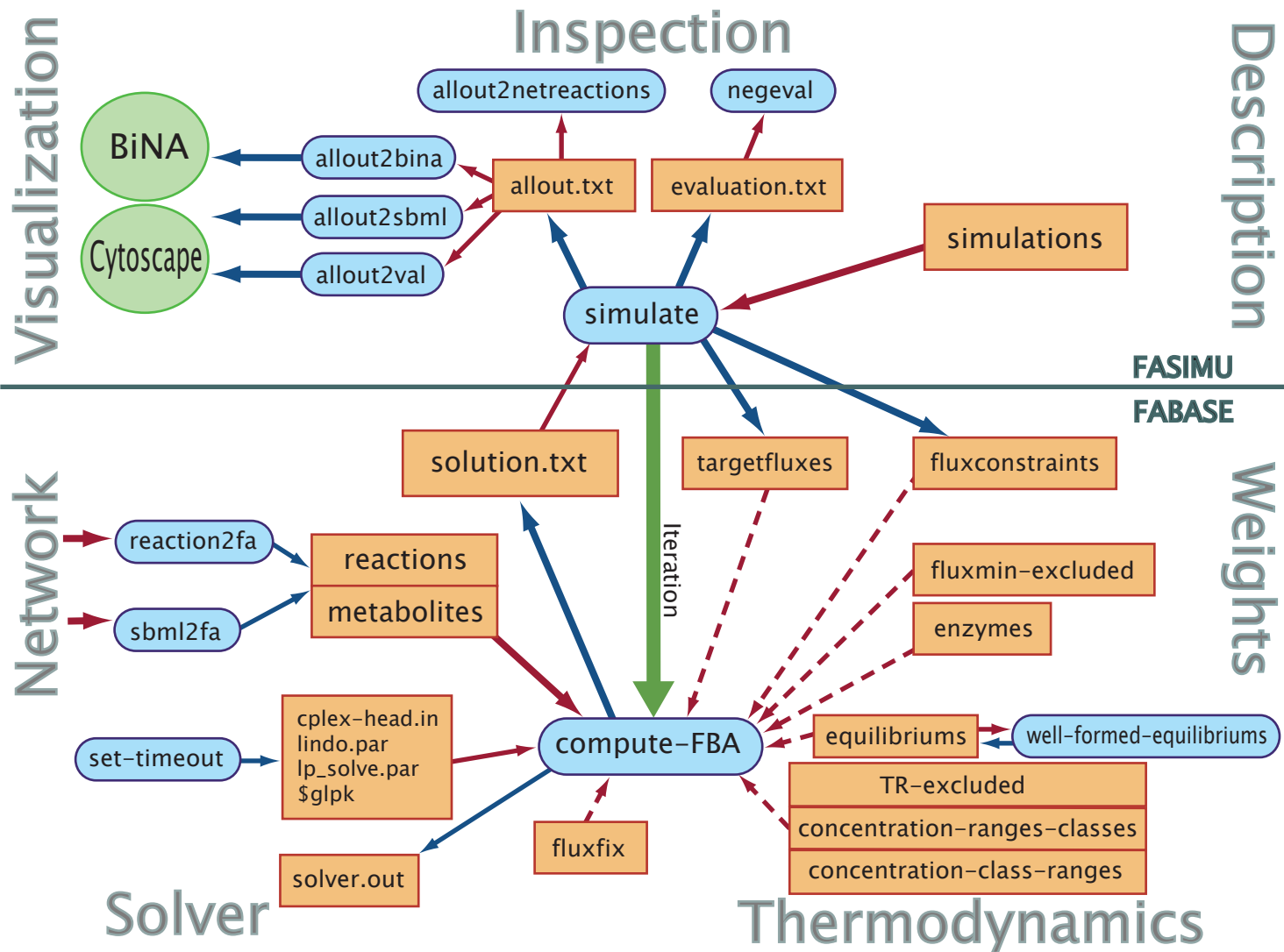

Supplement: Additional file 1 — FASIMU 2.2.1 release archive. This archive contains the complete FASIMU distributions and unzips in five directories: FASIMU contains the programs, FASIMU-Doc contains the documentation (manual and tutorial), FASIMU-Ery-Example contains a small example of the human erythrocyte, FASIM-Ecoli-Example a large example of the E. coli, FASIMU-Liver-Example another large example of the human hepatocyte. [file 1471-2105-12-28-S1.ZIP › FASIMU_Doc/FASIMU_Figure.pdf]
